# Indirect effects of the COVID-19 pandemic on malaria intervention coverage, morbidity, and mortality in Africa: a geospatial modelling analysis

**DOI:** 10.1016/S1473-3099(20)30700-3

**Published:** 2021-01

**Authors:** Daniel J Weiss, Amelia Bertozzi-Villa, Susan F Rumisha, Punam Amratia, Rohan Arambepola, Katherine E Battle, Ewan Cameron, Elisabeth Chestnutt, Harry S Gibson, Joseph Harris, Suzanne Keddie, Justin J Millar, Jennifer Rozier, Tasmin L Symons, Camilo Vargas-Ruiz, Simon I Hay, David L Smith, Pedro L Alonso, Abdisalan M Noor, Samir Bhatt, Peter W Gething

**Affiliations:** a Telethon Kids Institute, Perth Children's Hospital, Perth, WA, Australia; b Curtin University, Perth, WA, Australia; c Oxford Big Data Institute, Li Ka Shing Centre for Health Information and Discovery, Nuffield Department of Medicine, University of Oxford, Oxford, UK; d National Institute for Medical Research, Dar es Salaam, Tanzania; e Institute for Disease Modeling, Bellevue, WA, USA; f Institute for Health Metrics and Evaluation, University of Washington, Seattle, WA, USA; g Department of Health Metrics Sciences, School of Medicine, University of Washington, Seattle, WA, USA; h Global Malaria Programme, World Health Organization, Geneva, Switzerland; i Department of Infectious Disease Epidemiology, Imperial College London, London, UK

## Abstract

**Background:**

Substantial progress has been made in reducing the burden of malaria in Africa since 2000, but those gains could be jeopardised if the COVID-19 pandemic affects the availability of key malaria control interventions. The aim of this study was to evaluate plausible effects on malaria incidence and mortality under different levels of disruption to malaria control.

**Methods:**

Using an established set of spatiotemporal Bayesian geostatistical models, we generated geospatial estimates across malaria-endemic African countries of the clinical case incidence and mortality of malaria, incorporating an updated database of parasite rate surveys, insecticide-treated net (ITN) coverage, and effective treatment rates. We established a baseline estimate for the anticipated malaria burden in Africa in the absence of COVID-19-related disruptions, and repeated the analysis for nine hypothetical scenarios in which effective treatment with an antimalarial drug and distribution of ITNs (both through routine channels and mass campaigns) were reduced to varying extents.

**Findings:**

We estimated 215·2 (95% uncertainty interval 143·7–311·6) million cases and 386·4 (307·8–497·8) thousand deaths across malaria-endemic African countries in 2020 in our baseline scenario of undisrupted intervention coverage. With greater reductions in access to effective antimalarial drug treatment, our model predicted increasing numbers of cases and deaths: 224·1 (148·7–326·8) million cases and 487·9 (385·3–634·6) thousand deaths with a 25% reduction in antimalarial drug coverage; 233·1 (153·7–342·5) million cases and 597·4 (468·0–784·4) thousand deaths with a 50% reduction; and 242·3 (158·7–358·8) million cases and 715·2 (556·4–947·9) thousand deaths with a 75% reduction. Halting planned 2020 ITN mass distribution campaigns and reducing routine ITN distributions by 25%–75% also increased malaria burden to a total of 230·5 (151·6–343·3) million cases and 411·7 (322·8–545·5) thousand deaths with a 25% reduction; 232·8 (152·3–345·9) million cases and 415·5 (324·3–549·4) thousand deaths with a 50% reduction; and 234·0 (152·9–348·4) million cases and 417·6 (325·5–553·1) thousand deaths with a 75% reduction. When ITN coverage and antimalarial drug coverage were synchronously reduced, malaria burden increased to 240·5 (156·5–358·2) million cases and 520·9 (404·1–691·9) thousand deaths with a 25% reduction; 251·0 (162·2–377·0) million cases and 640·2 (492·0–856·7) thousand deaths with a 50% reduction; and 261·6 (167·7–396·8) million cases and 768·6 (586·1–1038·7) thousand deaths with a 75% reduction.

**Interpretation:**

Under pessimistic scenarios, COVID-19-related disruption to malaria control in Africa could almost double malaria mortality in 2020, and potentially lead to even greater increases in subsequent years. To avoid a reversal of two decades of progress against malaria, averting this public health disaster must remain an integrated priority alongside the response to COVID-19.

**Funding:**

Bill and Melinda Gates Foundation; Channel 7 Telethon Trust, Western Australia.

## Introduction

The ongoing COVID-19 pandemic is the most sustained, disruptive, and lethal infectious disease outbreak since the influenza pandemic of 1918. As of late August, 2020, the reported case incidence of COVID-19 in Africa remains modest compared with many regions worldwide; however, cases have been detected in most countries and incidence in some places is increasing rapidly. Countries in Africa are mounting a concerted public health response to limit the potential extent of COVID-19 morbidity and mortality on the continent,^1^, ^2^ drawing on decades of experience in large-scale public health activities to mitigate the burden of endemic and epidemic infectious diseases. Compared with the responses to COVID-19 in high-income nations, however, the measures taken in Africa come amid the backdrop of more acute health-system resource limitations^3^ and persistently higher morbidity and mortality from other infectious diseases.^4^, ^5^ As such, on top of concerns about the disease itself, alarm is growing about the broader health, economic, and societal effects of the measures imposed to slow the spread of COVID-19. Of particular concern is the possible disruption to efforts to control other endemic diseases that pose ongoing threats.

**Research in context**

**Evidence before this study**

Decades of data collection and recent modelling efforts have shown declining malaria burden in Africa since 2000. Past research by the Malaria Atlas Project, in collaboration with WHO, has been used to estimate the proportion of malaria cases that have been prevented by widespread use of antimalarial interventions. The effects of and the proposed response to the COVID-19 pandemic pose an immediate threat to distribution of insecticide-treated nets (ITNs) and access to effective treatment with antimalarial drugs, the two most important components of malaria control in Africa. We searched PubMed on June 24, 2020, using the search terms [“COVID-19” OR “SARS-CoV-2” OR “2019-nCoV” OR “coronavirus”] AND malaria, for articles published in English, with no date restrictions. Of the 97 articles returned, three were commentary or policy pieces that highlighted the possible indirect effect of the COVID-19 pandemic on malaria control and burden but did not include quantitative analyses. The remaining 94 articles were not relevant. Plausible effects of the COVID-19 pandemic on malaria interventions and the resulting implications for malaria burden have not previously been assessed through quantitative analyses.

**Added value of this study**

Using a range of plausible reduction scenarios for ITN distribution and provision of antimalarial drug treatment, we estimated the possible additional malaria-attributable morbidity and mortality across malaria-endemic African countries in 2020 relative to a baseline in which intervention deployment is unhindered. The regional-level and national-level results show geographically varying implications of reduced intervention coverage, reflecting differences in epidemiology and pre-pandemic intervention status within countries. For example, areas that had mass ITN distribution campaigns in 2019 were less affected in our scenarios because of the continued use of the nets already present in households.

**Implications of all the available evidence**

Decreased access to ITNs and antimalaria drugs, two key malaria interventions, would lead to potentially catastrophic increases in malaria morbidity and, in particular, mortality within Africa. By enumerating the potential outcomes of reduced coverage under a range of scenarios, we provide public health policy makers in malaria-endemic countries with additional information to consider when planning their COVID-19 responses. Where possible, to limit disease and death overall, future planning at international and national levels should consider the potential for catastrophic increases in malaria when designing COVID-19 mitigation strategies.

Since 2000, enormous progress has been made in reducing the burden of malaria in Africa.^6^, ^7^ The development of effective tools to reduce transmission by targeting the vector (such as the distribution of insecticide-treated nets (ITNs) and the spraying of structures with long-lasting insecticides) and the parasite (through reliable point-of-care diagnostic testing and effective antimalarial drugs), coupled with strengthening health systems and surveillance, have collectively saved millions of lives over the past decade.^7^ Until malaria transmission is interrupted altogether, however, these efforts merely suppress the disease. In the absence of a long-lasting vaccine, and because the underlying environmental suitability for *Plasmodium* spp transmission remains high,^8^ malaria is likely to resurge rapidly if intervention coverage falters.^9^, ^10^ This issue is especially pressing in places where levels of human population immunity are waning because of high intervention coverage.

The COVID-19 epidemic in Africa threatens malaria control in numerous ways. First, already-fragile health-system capacity risks being overwhelmed, meaning access to primary care for routine case management and the availability of hospitalisation for severe malaria could decrease sharply. Second, delivery of malaria control across the continent relies on a substantial workforce distributed across multiple sectors, including front-line health workers, health-system administrators, and logistic and field personnel orchestrating community-based intervention delivery. Widespread absenteeism due to illness, restrictions on movement, or diversion to the COVID-19 response means that this workforce could be critically disrupted. Third, supply chains that allow malaria control commodities such as drugs, bednets, rapid diagnostic test kits, and insecticide to be manufactured, procured, and delivered internationally and within Africa will be jeopardised by movement restrictions. Fourth, malaria control relies heavily on the decision making of patients and their families, including choosing to leave their homes to seek care for febrile children and receiving ITNs delivered at antenatal clinics or schools. Decisions such as these will be affected by additional illness, risk perception, or movement restrictions, all of which could jeopardise the provision and use of key malaria interventions.

Recognition of the threat posed to malaria control by COVID-19 has been widespread,^11^ and there is an urgent need to properly contextualise these threats amid rapidly evolving global health priorities. Doing so will require granular information with which to compare the relative threats posed by the spatially varying deterioration of malaria interventions. In this Article, we focus on the two primary tools for reducing malaria transmission and disease burden in Africa: ITNs and effective treatment with antimalarial drugs. We use the data and analytical architectures of the Malaria Atlas Project,^6^, ^7^, ^12^, ^13^ Global Burden of Disease Study (GBD),^14^ and World Malaria Report^15^ to evaluate scenarios of declining population coverage of these interventions and their consequences for case incidence and malaria mortality.

Concerns over the effect of COVID-19 on malaria in Africa prompted WHO to issue an urgent request for analyses characterising the magnitudes of potential resurgences in cases and deaths from malaria. This research was a direct response to this call for action, and our results have been incorporated into a newly released report^16^ that will help guide the global response to this crisis.

## Methods

### Overview

**Figure 1:**
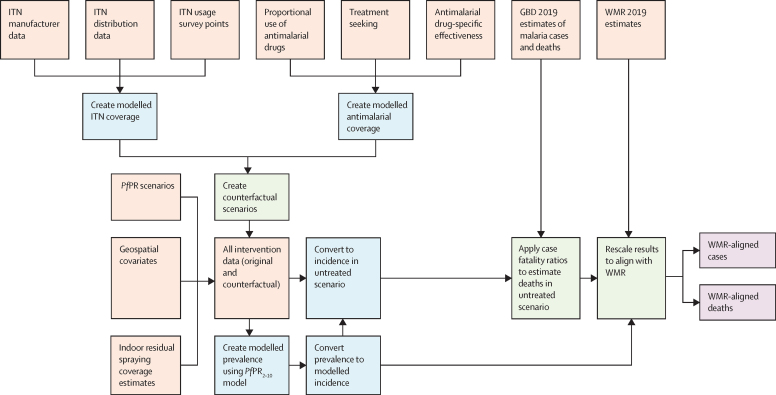
Simplified methodological flow chart Orange rectangles represent input datasets, some of which are modelled results from preceding analyses. Blue rectangles represent predefined process steps for which we supplied new data. Green rectangles are new conversions added to the production chain for this project. Purple rectangles are the results of the analysis. GBD=Global Burden of Disease Study. ITN=insecticide-treated net. *Pf*PR=*Plasmodium falciparum* parasite rate. WMR=World Malaria Report.

We analysed the potential effect of COVID-19 on malaria by combining the following components (figure 1): (1) up-to-date geospatial estimates of contemporary ITN and antimalarial drug coverages; (2) varied scenarios of deteriorating coverage for each intervention separately and in combination; (3) a Bayesian geostatistical space–time model to predict infection prevalence at a 5 × 5 km spatial resolution that incorporates ITN and antimalarial drug coverages as model covariates; (4) an established natural history model used to infer age-specific clinical incidence rates from malaria prevalence; and (5) an established geospatial model to predict malaria-attributable mortality given the incidence rate and effective treatment rates, calibrated to malaria-specific and an all-cause mortality envelopes provided by GBD 2019.^14^ The results of this analysis were aligned with those from the 2019 World Malaria Report^15^ to maximise the interpretability of the results and the comparability between our findings and established benchmarks. Our analysis included all malaria-endemic countries of Africa with the exception of Botswana, Comoros, Cape Verde, Djibouti, Eswatini, and São Tomé and Príncipe, where very low transmission and small numbers of cases and deaths meant analysis was not appropriate. We summarise each model component below, with further technical details provided in the appendix.

### ITN coverage and deterioration scenarios

Realistically estimating the public health effect of distributions on ITN coverage requires an explicit accounting of net procurement, distribution, and retention and use by households. We calculated ITN coverage (defined as the proportion of people sleeping under an ITN) for 2000–20 using an adaptation of an established mixed-modelling framework.^7^, ^17^ The model first uses a Gibbs sampler-based mechanistic model to triangulate data on ITN stock within countries, distributions, and ownership to produce estimates of country-specific ITN access and retention rates. These national-level indicators are then disaggregated spatially and converted to coverage via a series of spatiotemporal regressions. Models were calibrated to data on ITN stock from the Alliance for Malaria Prevention's Net Mapping Project,^18^ data on ITN distributions from national malaria control programmes and the African Leaders Malaria Alliance project, and data on ITN ownership from 156 nationally representative surveys conducted and disseminated by the Demographic and Health Surveys (DHS) Program.^19^ For projected 2020 distribution campaigns, we used data on planned timing and distribution size (number of nets) from the African Leaders Malaria Alliance and the President's Malaria Initiative Malaria Operational Plans.^20^

In almost every African country, ITNs are distributed on an ongoing basis via routine patient contacts with the health system, such as antenatal clinics and child immunisation centres, and sometimes additionally through schools. However, in most countries a larger proportion of ITNs are distributed directly to households via intermittent mass campaigns, usually every 2–4 years. An immediate risk is that mass campaigns planned for 2020 will be cancelled in light of the many challenges imposed by COVID-19 in Africa. Meanwhile, broader supply chain, health-system, and behavioural obstacles are likely to diminish the capacity of routine distribution channels to deliver ITNs to populations in need. As such, we explored three counterfactual scenarios whereby planned mass campaign distributions ceased for 2020, while routine channels were reduced by 25%, 50%, or 75% relative to the baseline scenario wherein all distributions proceeded as planned in 2020. In each scenario, we captured the fact that ITNs already present within households at the outset of 2020 will continue to offer waning protection in accordance with country-specific retention rates. Therefore, countries with large distributions in 2019 are likely to be less affected by service disruptions than countries that were planning mass campaigns in 2020.

### Antimalarial drug coverage and deterioration scenarios

We estimated the fraction of clinical malaria cases receiving effective treatment at a 5 × 5 km spatial resolution from 2000 to 2020. This model combines the fraction of children under 5 years of age who seek and receive treatment, the fractional use of antimalarial drug by antimalarial class, and the effectiveness of antimalarial drug by antimalarial class. Treatment seeking was modelled with use of an existing geospatial approach to estimate the fraction seeking care from national surveys on caregivers responses.^21^ This approach relied on the assumption that treatment seeking for fever in children under 5 years of age in malaria-endemic African countries adequately represents malaria-specific treatment seeking patterns for all age groups. Data on care-seeking behaviour and antimalarial drug access from DHS Program surveys,^19^ Malaria Indicator Surveys,^22^ and Multiple Indicator Cluster Surveys,^23^ were used in a generalised fractional regression model to estimate the country-year-specific fraction of malaria care seekers receiving different classes of antimalarial drugs. A geostatistical model was applied to site-specific clinical efficacy data compiled by the Worldwide Antimalarial Resistance Network^24^ to create drug-country-year-specific estimates of treatment effectiveness for those receiving antimalarials, while also incorporating reduction factors for drug quality and patient non-adherence to drug use protocols. These three components were combined to yield an overall estimate of the fraction of malaria cases receiving effective treatment. Because no data were yet available for 2020, we assumed the 2020 baseline scenario would correspond to coverage levels in 2019.

We then imposed three simple coverage reduction scenarios whereby the proportion of patients with malaria treated with antimalarial drugs was reduced 25%, 50%, or 75% from the baseline level. Because nearly all antimalarial drug access was by routine channels, there was no equivalent of a mass campaign to consider other than seasonal malaria chemoprevention, which we did not include in this analysis. We thus explored simple levels of reduction in antimalarial drug coverage, recognising that, in reality, deteriorations in access to treatment would occur in a highly spatially heterogenous way, reflecting local systems of drug supply and the varying effects of the COVID-19 outbreak.

### Spatiotemporal estimation of infection prevalence and intervention impact

A Bayesian geostatistical framework has been presented previously for estimating malaria infection prevalence and intervention impact.^7^ The updated response data consist of 53 770 observations of community-level *Plasmodium falciparum* parasite rate (*Pf*PR)—ie, the proportion of the population carrying the parasite—in Africa from 2000 to 2020. These observations result from parasitaemia tests conducted for 3·97 million individuals, age-standardised to 2–10-year-old children (*Pf*PR_2–10_),^25^ and are collated by the Malaria Atlas Project on an ongoing basis from the DHS Program, national Malaria Indicator Surveys, and the published literature, using systematic approaches described previously.^26^ *P falciparum* was the focus of this research because this species is responsible for the majority of malaria infections and deaths in Africa. The prevalence model generated annual realisations of *Pf*PR_2–10_ for each 5 × 5 km pixel across malaria-endemic Africa. By triangulating data on *Pf*PR, ITNs, and antimalarial drugs, along with a suite of environmental covariates,^27^ we estimated the impact of each intervention on *Pf*PR_2–10_ and inferred the pre-intervention transmission intensity. The fitted model was then used to predict a baseline *Pf*PR_2–10_ in 2020 using the pre-pandemic ITN and antimalarial drug coverage estimates, thus reflecting 2020 infection prevalence in the absence of COVID-19 disruptions. We then derived counterfactual versions for each of the scenarios of deteriorating ITN and antimalarial drug coverage. For each scenario, a set of 100 realisation surfaces was generated and propagated through the incidence and mortality models described below. Results were converted into population-weighted estimates by intersecting the realisations with gridded population surfaces. Finally, we summarised mean values and 95% uncertainty intervals (UIs) for each nation and for all of malaria-endemic Africa from the set of realisations, while also calculating percentage change estimates relative to the baseline means.

### Estimation of clinical incidence and malaria mortality

Each scenario of *Pf*PR_2–10_ in 2020 was used to generate a corresponding estimate of clinical incidence rate using an established natural history model.^28^ An existing approach^6^, ^13^ was then used to infer corresponding levels of malaria-attributable mortality for each clinical incidence scenario. The mortality estimation model relied on cause of death data and an all-cause mortality envelope, both of which were obtained from GBD 2019 data.^14^ This approach produces spatially heterogeneous surfaces of case fatality rate for untreated malaria for each modelled year. Untreated incidence was derived by multiplying the incidence surfaces by one minus antimalarial drug coverage, and the resulting grid was multiplied by the case fatality rate surface to yield a 5 × 5-km map of deaths due to malaria. All mortality estimates were proportionately rescaled to align our baseline values with the World Malaria Report estimates for the year 2018.^15^ The mortality estimation framework thus relied on the effective treatment with an antimalarial drug in two ways: as a covariate in the *Pf*PR_2–10_ model and for cleaving incidence into treated and untreated cases. Antimalarial drug coverage not only reduces incidence by reducing the duration that infected individuals contribute to onward transmission, but also reduces deaths by reducing the number of uncomplicated cases that develop severe, life-threatening malaria.

### Role of the funding source

The funder of the study had no role in study design, data collection, data analysis, data interpretation, or writing of the report. The corresponding author had full access to all the data in the study and had final responsibility for the decision to submit for publication.

## Results

**Figure 2:**
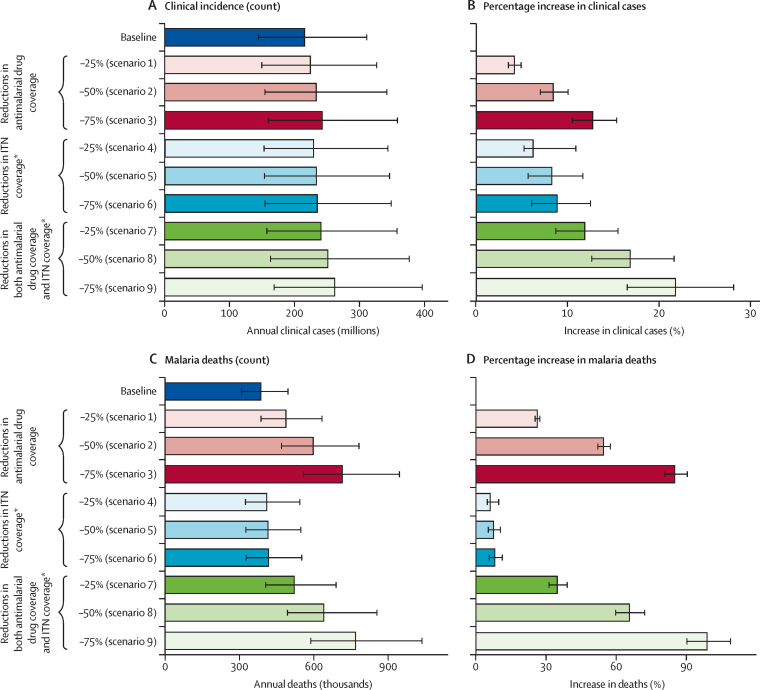
Estimated effect of deteriorating malaria control in Africa For each of nine scenarios of disrupted intervention coverage (table), we estimated the resulting number of malaria cases (A) and deaths (C), and the relative increases in cases (B) and deaths (D). Percentage reductions are relative to the baseline scenario of undisrupted antimalarial drug treatment and ITN distribution (as delivered via mass campaign and routine distributions). Error bars are 95% uncertainty intervals. ITN=insecticide-treated net. *ITN reduction scenarios consist of cessation of all mass distribution campaigns in addition to a reduction in routine distribution by the percentage specified.

The counterfactual analyses indicated that reducing the coverage of key malaria interventions would substantially affect the number of malaria cases in Africa relative to the baseline number of 215·2 (95% UI 143·7–311·6) million (figure 2A, B; table). Notably, reducing antimalarial drug coverage would have a greater impact on malaria incidence than decreasing ITN coverage. For example, in our model, a 75% drop in antimalarial drug coverage (scenario 3) increased the case count by 27·1 (22·2–32·8) million, whereas a 75% drop in routine ITN distributions coincident with a complete cessation of mass ITN distributions (scenario 6) increased cases by 18·8 (12·8–26·7) million. The combined effects of 75% reductions in both interventions (scenario 9) caused cases to increase by 46·4 (35·0–60·0) million.

**Table:** Baseline and counterfactual scenario estimates of malaria incidence and mortality for Africa

	**Scenario conditions**			**Cases**			**Deaths**		
	Mass ITN distribution	Reduction in routine ITN distribution, %	Reduction in antimalarial treatment, %	Total, millions	Increase from baseline, millions	Increase from baseline, %	Total, thousands	Increase from baseline, thousands	Increase from baseline, %
Baseline	Yes	..	..	215·2 (143·7–311·6)	..	..	386·4 (307·8–497·8)	..	..
Scenario 1	Yes	..	25%	224·2 (148·7–326·8)	8·9 (7·4–10·5)	4·1% (3·4–4·9)	487·9 (385·3–634·6)	101·5 (97·1–106·5)	26·3% (25·1–27·6)
Scenario 2	Yes	..	50%	233·1 (153·7–342·5)	17·9 (14·8–21·4)	8·3% (6·9–10·0)	597·4 (468·0–784·4)	211·0 (200·8–223·0)	54·6% (52·0–57·7)
Scenario 3	Yes	..	75%	242·3 (158·7–358·8)	27·1 (22·2–32·8)	12·6% (10·3–15·2)	715·2 (556·4–947·9)	328·7 (311·6–350·2)	85·1% (80·6–90·6)
Scenario 4	No	25%	..	228·4 (151·6–343·3)	13·2 (11·0–23·3)	6·1% (5·1–10·8)	410·0 (322·8–545·5)	23·6 (18·1–38·5)	6·1% (4·7–10·0)
Scenario 5	No	50%	..	232·8 (152·3–345·9)	17·6 (11·9–24·9)	8·2% (5·5–11·6)	415·5 (324·3–549·4)	29·1 (19·8–41·3)	7·5% (5·1–10·7)
Scenario 6	No	75%	..	234·0 (152·9–348·4)	18·8 (12·8–26·7)	8·7% (5·9–12·4)	417·6 (325·5–553·1)	31·1 (21·2–44·3)	8·1% (5·5–11·5)
Scenario 7	No	25%	25%	240·5 (156·5–358·2)	25·2 (18·4–33·1)	11·7% (8·6–15·4)	520·9 (404·0–691·9)	134·5 (120·1–151·7)	34·8% (31·1–39·2)
Scenario 8	No	50%	50%	251·0 (162·2–377·0)	35·8 (26·8–46·1)	16·6% (12·4–21·4)	640·2 (492·0–856·7)	253·7 (230·3–279·8)	65·7% (59·6–72·4)
Scenario 9	No	75%	75%	261·6 (167·7–396·8)	46·4 (35·0–60·0)	21·5% (16·3–27·9)	768·6 (586·1–1038·7)	382·1 (348·2–421·5)	98·9% (90·1–109·1)

Data in parentheses are 95% uncertainty intervals. All uncertainty estimates were derived at the pixel level from the set of realisations and then summarised for all malaria-endemic countries in Africa. Estimates are also represented graphically in figure 2.

The potential increase in malaria-attributable deaths as a result of the COVID-19 pandemic is a greater cause for concern than the potential increase in malaria cases. At baseline, with undisrupted intervention, we estimated 386·4 (307·8–497·8) thousand malaria-attributable deaths in 2020. Although the modelled reductions in ITN coverage (scenarios 4–6) led to proportional increases in both cases and deaths, the effect of decreasing antimalarial drug coverage was more pronounced because of the role that effective treatment of malaria plays in preventing deaths from the disease (figure 2C, D; table). For example, even if ITN distributions in 2020 were unaffected by COVID-19, we estimated that a 75% reduction in antimalarial drug coverage (scenario 3) would increase deaths from malaria by 328·7 (311·6–350·2) thousand. Furthermore, the additive effect of reductions in both interventions (scenarios 7–9), which increased cases and also treated fewer of them, increased the number of deaths by 134·5 (120·1–151·7) thousand with 25% reductions in both interventions, 253·7 (230·3–279·8) thousand with 50% reductions, and 382·1 (348·2–421·5) thousand with 75% reductions.

**Figure 3:**
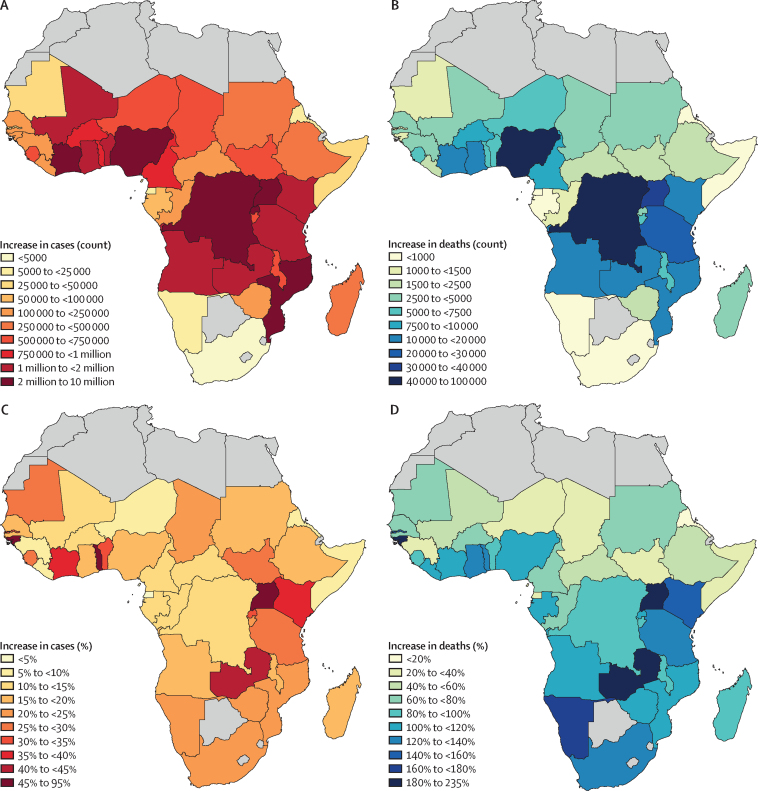
Estimated effects of a 75% reduction in malaria control in Africa by country Estimates are shown for scenario 9 (no mass distributions of insecticide-treated nets, and 75% reductions in routine insecticide-treated net distribution and antimalarial drug treatment, relative to undisrupted levels). Results are mapped for absolute increases in cases (A) and deaths (B), and relative increases in cases (C) and deaths (D). Countries shaded in grey were not included in the analysis.

**Figure 4:**
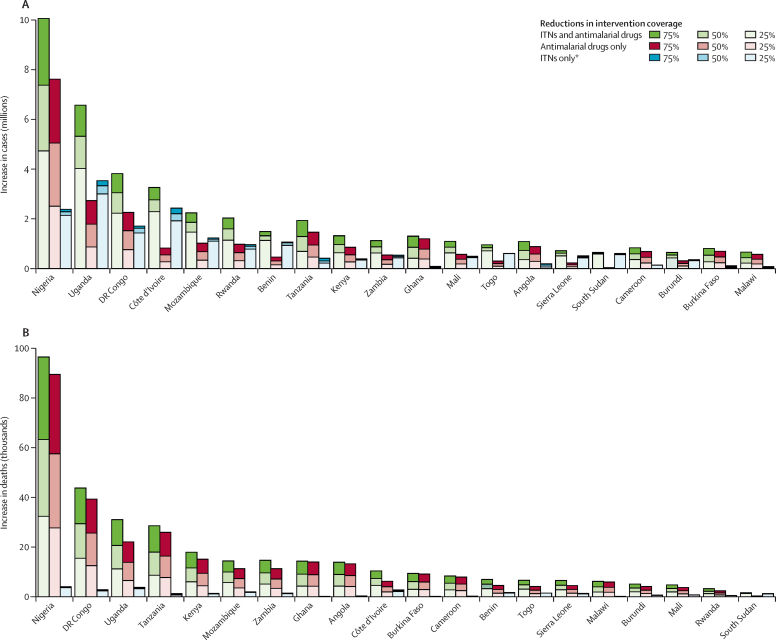
Estimated effects of deteriorating malaria control by intervention type and disruption level for the 20 most affected countries Estimated increases in cases (A) and deaths (B) for each country given reductions of 25%, 50%, and 75% (*vs* undisrupted baseline levels) in ITN distribution and antimalarial drug treatment, separately and combined. Bars are cumulative such that bottom segment (lightest shading) represents a 25% reduction, the bottom two segment (light and intermediate shading) represent a 50% reduction, and the full height of the bar (light, intermediate, and dark shading) represent a 75% reduction. ITN=insecticide-treated net. *ITN reduction scenarios consist of cessation of all mass distribution campaigns in addition to a reduction in routine distribution by the percentage specified.

Malaria burden, intervention coverages, and case fatality rate varied spatially and were thus estimated using geospatial modelling approaches. This methodological framework produced maps of malaria incidence and mortality with 5 × 5 km spatial resolution for each of the counterfactual scenarios. To better illustrate the effects of national-level policy decisions in response to COVID-19, we summarised and mapped outputs by country (Figure 3, Figure 4). These national-level results present a more nuanced picture than those at continental scale. Geographical patterns in the absolute effects on morbidity and mortality (figure 3 A, B) largely reflect the magnitude of underlying burden, such that countries with high transmission and high population—including Nigeria, DR Congo, and Mozambique—are predicted to yield the largest increases should malaria control falter. Patterns of relative impact (figure 3 C, D) are different, being additionally driven by current ITN and antimalarial drug coverage levels, variations in case fatality rate, and whether or not countries had a mass ITN distribution campaign scheduled for 2020. Figure 4 gives additional insight into the relative roles of disruption to different interventions in each country, particularly for the predicted effects on cases. Of the four countries with the largest predicted effects on cases, Nigeria and DR Congo are predicted to be the most impacted by disruptions to antimalarial drugs, whereas in Uganda and Côte d'Ivoire, disruptions to ITN distributions are more impactful. These differences reflect both the underlying transmission setting and the current status of those interventions in each country.

**Figure 5:**
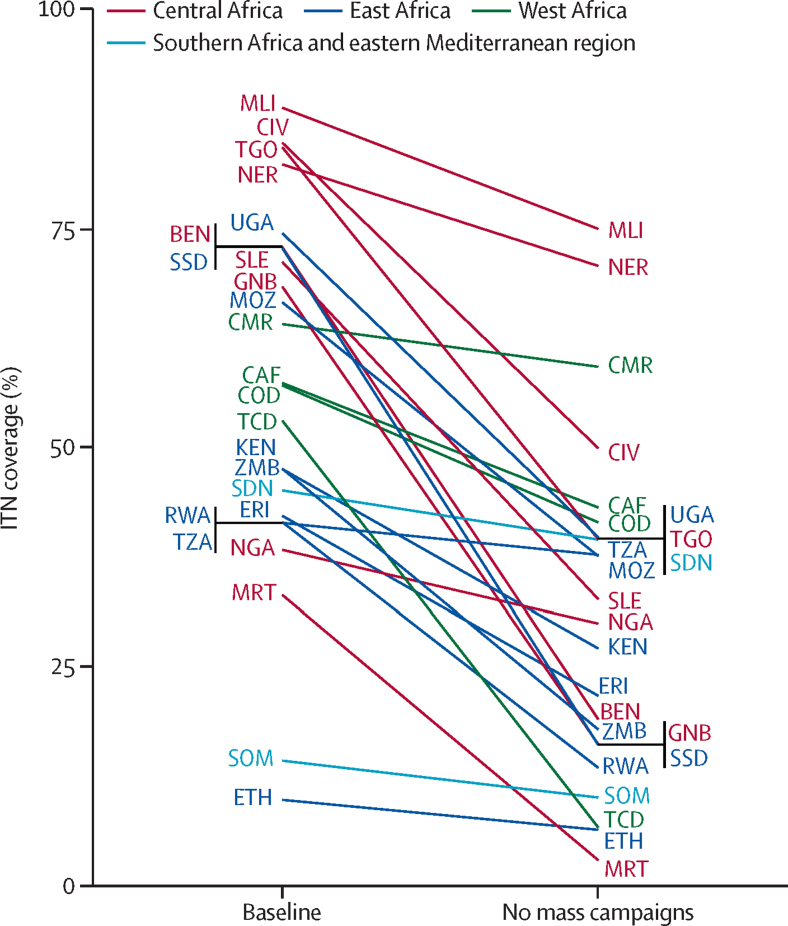
Effect of cancellation of mass distribution campaigns on ITN coverage Shown, for the 24 countries with scheduled mass campaigns in 2020, are estimated ITN coverage rates in two scenarios: one in which all 2020 mass and routine distribution campaigns continue as planned (the baseline scenario) and the other in which mass campaigns are cancelled, showing the difference in expected coverage rates due to the suspension of mass campaigns alone. Details of the size of the mass campaigns planned in 2020 for each country are shown in the appendix (p 1). BEN=Benin. CMR=Cameroon. CAF=Central African Republic. TCD=Chad. CIV=Côte d'Ivoire. COD=Democratic Republic of the Congo. ERI=Eritrea. ETH=Ethiopia. GNB=Guinea‐Bissau. ITN=insecticide-treated net. KEN=Kenya. MLI=Mali. MRT=Mauritania. MOZ=Mozambique. NER=Niger. NGA=Nigeria. RWA=Rwanda. SLE=Sierra Leone. SOM=Somalia. SSD=South Sudan. SDN=Sudan. TZA=Tanzania. TGO=Togo. UGA=Uganda. ZMB=Zambia.

Cancelling mass ITN campaigns would have a substantial impact on 2020 ITN coverage in Africa, especially in those countries that have not had mass campaigns in several years (figure 5). South Sudan, for example, is scheduled to have its largest mass campaign in history in 2020, increasing ITN coverage from 32·5% in 2019 to 72·7% in 2020. In the absence of mass campaigns, 2020 coverage is instead projected to decline to 16·0%, falling additionally to 12·3% if routine channels suffer a 75% decrease in capacity. South Sudan is one of the few countries in which reductions in ITN coverage alone are likely to have a larger impact than reductions in antimalarial drug coverage alone, with no 2020 mass campaign and a 25% reduction in routine ITN distribution causing an increase of 564·0 (277·1–968·1) thousand cases and 1167 (573–2003) deaths in our model. By contrast, a 25% reduction in antimalarial drug coverage would increase cases by 13·0 (7·8–17·2) thousand and deaths by 105 (94–113). Other countries likely to be affected more by reductions in ITN distribution than by declines in antimalarial drug coverage include Benin, Côte d'Ivoire, Guinea-Bissau, and Chad, all of which have ambitious mass ITN campaigns planned for 2020. Tables of planned national-level ITN distributions and counterfactual results are provided in the appendix (pp 1–15).

## Discussion

The intensity of COVID-19 outbreaks in African countries and the resulting effects on malaria incidence and mortality are inherently unpredictable. It is inevitable, however, that the COVID-19 pandemic will pose severe challenges to programmes that supply the key interventions responsible for reducing malaria morbidity and mortality. In this study, we provide an evaluation of these effects under a plausible range of scenarios. A key assumption of this research is that malaria morbidity and mortality are not directly affected by COVID-19 infection, as it is currently unknown what effects co-infection with malaria will have on either disease.

In our worst-case scenario, in which mass ITN distributions are cancelled and there are 75% reductions in routine ITN distribution and effective treatment of cases with an antimalarial, the incidence of malaria in 2020 would increase by 21·5% (95% UI 16·3–27·9), or 46·4 (35·0–60·0) million cases, relative to the mean baseline level across malaria-endemic African countries. Furthermore, in this scenario, malaria-attributable deaths would nearly double, from 386·4 (307·8–497·8) thousand to 768·6 (586·1–1038·7) thousand. The large increase in deaths is a result of the crucial role of antimalarial drugs in preventing progression to death in malaria-infected individuals. As such, if COVID-19 outbreaks lead to fewer malaria cases being effectively treated, whether through shifts in public health policy or altered individual behaviours, we predict a large increase in malaria mortality. This accentuated role of antimalarial drugs in our approach stems from our application of the case fatality rate to only untreated cases, meaning we assume that cases left untreated result in a proportionate increase in deaths. Alternate assessments of the effect of COVID-19 on malaria in Africa^29^ that arrived at a contradictory conclusion did not use this assumption.

The smaller effect of decreasing ITN distributions, as opposed to decreasing antimalarial drugs, on malaria incidence differs from previous research that showed ITNs to be largely responsible for driving down malaria in Africa since 2000.^7^ This difference is interpretable as an effect of the existing stock of ITNs held by households continuing to provide effective, albeit waning, protection against malaria transmission. Our modelled ITN estimates, which predict waning coverage rather than a dramatic drop, differ from those used to derive alternate assessments of malaria incidence in Africa in 2020.^29^ By comparing results between countries, our analysis also suggests that disruptions to mass ITN distribution campaigns will have a larger effect on malaria than disruptions in routine ITN distributions, which reflects the larger role of mass campaigns in maintaining coverage. Furthermore, if COVID-19 causes a widespread decline in ITN distributions in 2020, the full impact of increased malaria incidence will not be realised until future years.

The effects of COVID-19 on malaria vary widely between nations, and are driven by each country's epidemiological context, size, and current intervention coverage status. In Guinea-Bissau, for example, we predict an increase of 217·8 (110·5–318·7) thousand in the number of cases in the worst-case scenario, which reflects the role of ITNs and antimalarial drugs in decreasing interventions in this nation. This pattern is repeated elsewhere and is intuitive, with countries planning ITN mass campaigns in 2020 attributing a greater proportion of their increased cases to ITNs than countries that had recent mass ITN distributions. Similarly, malaria-endemic countries that have high coverage of antimalarial drugs in the absence of COVID-19-related disruptions will have larger increases in cases and deaths within our hypothetical scenarios. Unsurprisingly, the largest absolute effects are predicted in the countries with the greatest malaria burden, such as Nigeria and DR Congo, which have large populations and high environmental suitability for malaria transmission. In the most extreme scenario, Nigeria would have estimated increases of 10·1 (7·3–13·0) million cases and 96·5 (87·7–105·1) thousand deaths, and DR Congo would have increases of 3·8 (2·2–6·2) million cases and 43·8 (39·4–50·7) thousand deaths.

Any forward-looking analysis relies on multiple assumptions and simplifications. For example, we were unable to robustly estimate the proportion of patients with severe malaria who would receive effective inpatient care under normal circumstances, nor how hospital-based case management would be affected during this pandemic. As such, we focused our case-management scenarios on the effects on front-line treatment of uncomplicated cases with antimalarial drugs, but assumed that case fatality rates among untreated patients would remain unchanged. This assumption is probably a conservative one, because the survival rates of patients with severe malaria are likely to decline, further adding to the effects on mortality. Another important limitation of this research is that we do not address other malaria control interventions, such as intermittent or seasonal preventive treatment with chemoprophylaxis or indoor spraying of long-lasting insecticidal residues. Although these interventions are important in some contexts, their application is highly localised and thus their importance at continental or national scales is typically secondary to that of ITNs and antimalarial drugs. Likewise, we did not consider novel mitigation measures that might emerge in response to this crisis. For example, official guidelines now advocate a range of practical measures that malaria programmes can undertake to attempt to reduce surges in malaria burden.^30^ Lastly, this analysis ignores existing national plans to use risk-stratification strategies for optimising the control and treatment of malaria based on the level of burden. Such strategies could mitigate the increases we estimated by maintaining ITN and antimalarial drug coverage in areas with high levels of endemic malaria if supplies of these commodities became limited.

The COVID-19 pandemic has exerted enormous pressure on health systems in well resourced, high-income nations worldwide. Although many malaria-endemic African nations have shown remarkable resilience and adaptivity in the face of previous global health threats,^31^ they nevertheless face the unprecedented challenge of COVID-19 with a comparatively lower health-care system capacity and a higher baseline level of malaria burden. Our analysis suggests that the direct response to COVID-19 must be integrated with efforts to ensure malaria control is maintained. Failure to do so risks amplifying the mortality caused by this pandemic, especially in children, and reversing of one of the most impactful public health campaigns of the past two decades.

## Data sharing

All national-level results are available within the supplementary material. High resolution maps are available upon request from the Malaria Atlas Project.
