# Supplementary material for: Indirect effects of the COVID-19 pandemic on malaria intervention coverage, morbidity, and mortality in Africa: a geospatial modelling analysis
Source: Lancet Infect Dis. 2021 Jan;21(1):59–69. doi: 10.1016/S1473-3099(20)30700-3 (PMC7505634; doi:10.1016/S1473-3099(20)30700-3)
Supplement: Supplementary appendix [file mmc1.pdf]

THE LANCET
Infectious Diseases

Supplementary appendix

This appendix formed part of the original submission and has been peer reviewed. We post it as supplied by the authors.

Supplement to: Weiss DJ, Bertozzi-Villa A, RumishaSF, et al. Indirect effects of the COVID-19 pandemic on malaria intervention coverage, morbidity, and mortality in Africa: a geospatial modelling analysis. *Lancet Infect Dis* 2020; published online Sept 21. https://doi.org/10.1016/S1473-3099(20)30700-3.

Appendix

Associated manuscript: Indirect impacts of the COVID-19 pandemic on malaria intervention coverage, morbidity and mortality in sub-Saharan Africa: a geospatial modelling analysis

Table S1. The sizes of the scheduled mass ITN campaign for countries in sub-Saharan Africa in 2020. Millions of long-lasting insecticide-treated bednets per country.

Name	ISO3	Mass ITN distribution campaign size (millions)
Benin	BEN	8.25
Cameroon	CMR	2.75
Central African Republic	CAF	0.91
Chad	TCD	9.12
Côte d'Ivoire	CIV	17.1
Democratic Republic of the Congo	COD	20.4
Eritrea	ERI	1.9
Ethiopia	ETH	5.8
Guinea-Bissau	GNB	1.23
Kenya	KEN	13.4
Mali	MLI	6.55
Mauritania	MRT	1.45
Mozambique	MOZ	10.5
Niger	NER	6.17
Nigeria	NGA	14.8
Rwanda	RWA	6.31
Sierra Leone	SLE	3.97
Somalia	SOM	0.71
South Sudan	SSD	6.95
Sudan	SDN	2.87
Tanzania	TZA	2.99
Togo	TGO	5.96
Uganda	UGA	23.8
Zambia	ZMB	7.28

Table S2. National-level estimates for case and death counts and percentage increases. All estimates are aligned with year 2018 malaria population at risk, cases, and deaths as reported in the 2019 World Malaria Report. Note that due to the estimates both the baseline and counterfactual scenarios were derived from a set of realizations, which resulted in several instances where the scenario estimates were slightly lower than those from the baseline.

Name	Scenario	Cases (millions)	Increased cases (%)	Deaths (thousands)	Increased deaths (%)
Sub-Saharan Africa	Baseline	215.22 (143.71-311.6)	0% (0-0)	386.44 (307.78-497.77)	0% (0-0)
Sub-Saharan Africa	AM 25% Reduction	224.08 (148.67-326.78)	4.12% (3.43-4.9)	487.9 (385.27-634.62)	26.25% (25.13-27.55)
Sub-Saharan Africa	AM 50% Reduction	233.11 (153.65-342.48)	8.31% (6.87-9.96)	597.41 (468.05-784.4)	54.59% (51.97-57.7)
Sub-Saharan Africa	AM 75% Reduction	242.28 (158.66-358.78)	12.57% (10.33-15.23)	715.19 (556.43-947.88)	85.07% (80.64-90.62)
Sub-Saharan Africa	ITN 25% Reduction	228.43 (151.55-343.28)	6.14% (5.11-10.81)	410.03 (322.8-545.49)	6.1% (4.68-9.96)
Sub-Saharan Africa	ITN 50% Reduction	232.8 (152.29-345.94)	8.17% (5.55-11.57)	415.52 (324.27-549.4)	7.53% (5.11-10.69)
Sub-Saharan Africa	ITN 75% Reduction	234.03 (152.89-348.37)	8.74% (5.94-12.39)	417.59 (325.47-553.09)	8.06% (5.48-11.47)
Sub-Saharan Africa	Both 25% Reduction	240.46 (156.53-358.17)	11.72% (8.56-15.36)	520.93 (404.08-691.93)	34.8% (31.09-39.25)
Sub-Saharan Africa	Both 50% Reduction	251.03 (162.15-376.96)	16.64% (12.43-21.43)	640.17 (492.03-856.69)	65.66% (59.59-72.41)
Sub-Saharan Africa	Both 75% Reduction	261.58 (167.73-396.79)	21.54% (16.27-27.86)	768.59 (586.12-1038.73)	98.89% (90.09-109.08)
Angola	Baseline	7.05 (5.26-9.23)	0% (0-0)	13.43 (10.2-18.8)	0% (0-0)
Angola	AM 25% Reduction	7.35 (5.42-9.7)	4.15% (2.98-5.15)	17.55 (13.18-24.81)	30.7% (29.18-31.98)
Angola	AM 50% Reduction	7.64 (5.57-10.19)	8.34% (5.92-10.48)	21.96 (16.3-31.37)	63.59% (59.78-66.86)
Angola	AM 75% Reduction	7.94 (5.73-10.7)	12.56% (8.82-15.98)	26.67 (19.56-38.5)	98.67% (91.72-104.77)
Angola	ITN 25% Reduction	7.12 (5.29-9.35)	0.94% (0.62-1.39)	13.55 (10.26-19.06)	0.94% (0.62-1.4)
Angola	ITN 50% Reduction	7.2 (5.33-9.5)	2.06% (1.34-2.97)	13.7 (10.34-19.36)	2.06% (1.34-2.98)
Angola	ITN 75% Reduction	7.25 (5.36-9.6)	2.79% (1.82-4.03)	13.8 (10.39-19.56)	2.8% (1.82-4.05)
Angola	Both 25% Reduction	7.41 (5.45-9.83)	5.12% (3.58-6.57)	17.71 (13.26-25.14)	31.92% (29.96-33.7)
Angola	Both 50% Reduction	7.79 (5.64-10.48)	10.42% (7.16-13.61)	22.39 (16.5-32.24)	66.74% (61.76-71.46)
Angola	Both 75% Reduction	8.14 (5.81-11.1)	15.42% (10.46-20.36)	27.35 (19.88-39.91)	103.71% (94.89-112.3)
Benin	Baseline	4.44 (3.49-5.56)	0% (0-0)	7.08 (6.37-7.87)	0% (0-0)
Benin	AM 25% Reduction	4.59 (3.56-5.81)	3.45% (2.14-4.59)	8.51 (7.56-9.57)	20.22% (18.71-21.56)
Benin	AM 50% Reduction	4.74 (3.64-6.08)	6.93% (4.19-9.5)	10.03 (8.79-11.41)	41.62% (37.98-45.02)
Benin	AM 75% Reduction	4.9 (3.7-6.37)	10.45% (6.12-14.73)	11.63 (10.05-13.42)	64.18% (57.74-70.55)
Benin	ITN 25% Reduction	5.37 (3.86-7.64)	21.13% (10.6-37.5)	8.58 (7.05-10.82)	21.14% (10.61-37.51)
Benin	ITN 50% Reduction	5.47 (3.87-7.74)	23.36% (11.05-39.22)	8.74 (7.07-10.96)	23.37% (11.06-39.23)
Benin	ITN 75% Reduction	5.5 (3.88-7.8)	24.04% (11.32-40.41)	8.78 (7.09-11.05)	24.04% (11.32-40.41)
Benin	Both 25% Reduction	5.57 (3.91-7.89)	25.64% (12.13-42.09)	10.34 (8.3-13)	46.02% (30.32-65.14)
Benin	Both 50% Reduction	5.75 (3.96-8.26)	29.74% (13.64-48.62)	12.17 (9.59-15.49)	71.83% (50.5-96.82)
Benin	Both 75% Reduction	5.92 (4.01-8.59)	33.4% (14.91-54.57)	14.04 (10.88-18.08)	98.3% (70.82-129.77)

Name	Scenario	Cases (millions)	Increased cases (%)	Deaths (thousands)	Increased deaths (%)
Burkina Faso	Baseline	7.88 (5.55-10.96)	0% (0-0)	12.73 (9.86-16.7)	0% (0-0)
Burkina Faso	AM 25% Reduction	8.11 (5.62-11.46)	2.99% (1.24-4.54)	15.63 (11.9-20.82)	22.8% (20.71-24.66)
Burkina Faso	AM 50% Reduction	8.34 (5.69-11.97)	5.95% (2.42-9.21)	18.67 (13.98-25.26)	46.73% (41.81-51.24)
Burkina Faso	AM 75% Reduction	8.58 (5.75-12.49)	8.89% (3.54-13.99)	21.85 (16.1-30.03)	71.75% (63.28-79.81)
Burkina Faso	ITN 25% Reduction	7.91 (5.56-11.06)	0.49% (0.18-0.9)	12.79 (9.88-16.85)	0.49% (0.18-0.9)
Burkina Faso	ITN 50% Reduction	7.95 (5.57-11.14)	0.92% (0.35-1.65)	12.84 (9.89-16.98)	0.92% (0.35-1.65)
Burkina Faso	ITN 75% Reduction	7.99 (5.58-11.24)	1.43% (0.55-2.58)	12.91 (9.91-17.13)	1.44% (0.55-2.58)
Burkina Faso	Both 25% Reduction	8.15 (5.63-11.55)	3.49% (1.42-5.41)	15.7 (11.92-20.99)	23.4% (20.93-25.7)
Burkina Faso	Both 50% Reduction	8.42 (5.7-12.14)	6.86% (2.73-10.78)	18.83 (14.03-25.63)	47.98% (42.25-53.46)
Burkina Faso	Both 75% Reduction	8.68 (5.77-12.77)	10.28% (4-16.49)	22.13 (16.17-30.7)	73.94% (64.01-83.81)
Burundi	Baseline	2.8 (2.08-3.68)	0% (0-0)	5.12 (4.41-6.17)	0% (0-0)
Burundi	AM 25% Reduction	2.9 (2.12-3.88)	3.76% (2.07-5.51)	6.41 (5.43-7.86)	25.23% (23.2-27.35)
Burundi	AM 50% Reduction	3.01 (2.16-4.1)	7.52% (3.98-11.43)	7.78 (6.48-9.72)	52.02% (47.03-57.54)
Burundi	AM 75% Reduction	3.11 (2.2-4.33)	11.26% (5.69-17.68)	9.23 (7.56-11.77)	80.34% (71.37-90.71)
Burundi	ITN 25% Reduction	3.12 (2.17-4.47)	11.48% (4.57-21.36)	5.71 (4.61-7.49)	11.47% (4.56-21.36)
Burundi	ITN 50% Reduction	3.14 (2.18-4.52)	12.39% (5.06-22.73)	5.75 (4.63-7.57)	12.39% (5.05-22.74)
Burundi	ITN 75% Reduction	3.15 (2.19-4.54)	12.69% (5.18-23.35)	5.77 (4.64-7.61)	12.69% (5.17-23.36)
Burundi	Both 25% Reduction	3.23 (2.21-4.72)	15.33% (6.39-28.32)	7.12 (5.66-9.56)	39.19% (28.41-54.88)
Burundi	Both 50% Reduction	3.34 (2.25-5.05)	19.54% (8.08-37.2)	8.65 (6.74-11.97)	69.02% (52.83-93.99)
Burundi	Both 75% Reduction	3.45 (2.28-5.35)	23.28% (9.49-45.43)	10.23 (7.83-14.54)	99.82% (77.49-135.73)
Cameroon	Baseline	6.23 (3.64-9.83)	0% (0-0)	11.19 (9.36-13.5)	0% (0-0)
Cameroon	AM 25% Reduction	6.45 (3.72-10.3)	3.62% (2.14-4.79)	13.68 (11.28-16.69)	22.26% (20.46-23.64)
Cameroon	AM 50% Reduction	6.68 (3.8-10.79)	7.3% (4.25-9.73)	16.33 (13.26-20.14)	45.89% (41.66-49.2)
Cameroon	AM 75% Reduction	6.91 (3.87-11.29)	11.02% (6.34-14.8)	19.13 (15.31-23.86)	70.91% (63.57-76.78)
Cameroon	ITN 25% Reduction	6.37 (3.68-10.16)	2.28% (0.95-3.34)	11.45 (9.45-13.95)	2.29% (0.95-3.36)
Cameroon	ITN 50% Reduction	6.37 (3.68-10.16)	2.23% (1.08-3.39)	11.44 (9.46-13.96)	2.25% (1.08-3.4)
Cameroon	ITN 75% Reduction	6.37 (3.68-10.18)	2.33% (1.12-3.53)	11.45 (9.47-13.98)	2.34% (1.13-3.55)
Cameroon	Both 25% Reduction	6.59 (3.76-10.6)	5.85% (3.29-7.84)	13.98 (11.4-17.18)	24.9% (21.83-27.23)
Cameroon	Both 50% Reduction	6.82 (3.84-11.1)	9.58% (5.36-12.88)	16.68 (13.4-20.72)	48.99% (43.18-53.47)
Cameroon	Both 75% Reduction	7.06 (3.91-11.62)	13.41% (7.35-18.17)	19.54 (15.46-24.56)	74.59% (65.17-81.9)
Central African Republic	Baseline	1.62 (1.08-2.36)	0% (0-0)	3.65 (2.41-5.73)	0% (0-0)
Central African Republic	AM 25% Reduction	1.65 (1.09-2.43)	1.83% (0.92-2.77)	4.06 (2.66-6.43)	11.19% (10.19-12.21)
Central African Republic	AM 50% Reduction	1.68 (1.1-2.49)	3.65% (1.8-5.61)	4.48 (2.9-7.16)	22.7% (20.51-25)

Name	Scenario	Cases (millions)	Increased cases (%)	Deaths (thousands)	Increased deaths (%)
Central African Republic	AM 75% Reduction	1.71 (1.11-2.56)	5.47% (2.66-8.5)	4.92 (3.16-7.93)	34.53% (30.96-38.38)
Central African Republic	ITN 25% Reduction	1.71 (1.1-2.63)	5.71% (2.09-11.39)	3.86 (2.46-6.38)	5.71% (2.09-11.39)
Central African Republic	ITN 50% Reduction	1.72 (1.1-2.64)	6.35% (2.45-12.03)	3.89 (2.47-6.42)	6.35% (2.45-12.03)
Central African Republic	ITN 75% Reduction	1.72 (1.1-2.63)	5.91% (2.29-11.18)	3.87 (2.47-6.37)	5.91% (2.29-11.19)
Central African Republic	Both 25% Reduction	1.75 (1.11-2.69)	7.8% (3.15-14.09)	4.3 (2.71-7.14)	17.71% (12.63-24.56)
Central African Republic	Both 50% Reduction	1.78 (1.12-2.77)	9.89% (4.08-17.47)	4.75 (2.97-7.97)	30.08% (23.2-39.04)
Central African Republic	Both 75% Reduction	1.8 (1.13-2.82)	11.23% (4.71-19.47)	5.18 (3.22-8.73)	41.88% (33.55-52.38)
Chad	Baseline	2.52 (1.25-4.59)	0% (0-0)	8.69 (7.37-10.3)	0% (0-0)
Chad	AM 25% Reduction	2.57 (1.27-4.71)	1.99% (1.39-2.56)	9.31 (7.84-11.09)	7.04% (6.37-7.69)
Chad	AM 50% Reduction	2.62 (1.29-4.83)	4.01% (2.78-5.17)	9.94 (8.32-11.92)	14.31% (12.89-15.69)
Chad	AM 75% Reduction	2.68 (1.31-4.95)	6.05% (4.16-7.83)	10.59 (8.81-12.77)	21.81% (19.55-24.01)
Chad	ITN 25% Reduction	2.88 (1.34-5.63)	14.09% (6.65-22.55)	9.92 (7.86-12.63)	14.1% (6.64-22.59)
Chad	ITN 50% Reduction	2.9 (1.35-5.67)	14.81% (7.87-23.37)	9.98 (7.95-12.71)	14.82% (7.87-23.41)
Chad	ITN 75% Reduction	2.9 (1.35-5.68)	14.96% (7.94-23.61)	9.99 (7.96-12.74)	14.97% (7.94-23.66)
Chad	Both 25% Reduction	2.94 (1.37-5.75)	16.44% (8.97-25.19)	10.62 (8.43-13.54)	22.22% (14.34-31.48)
Chad	Both 50% Reduction	3.01 (1.39-5.91)	19.13% (10.59-28.71)	11.38 (8.95-14.59)	30.93% (21.49-41.61)
Chad	Both 75% Reduction	3.07 (1.4-6.05)	21.48% (12.02-31.7)	12.13 (9.48-15.6)	39.54% (28.58-51.47)
Congo	Baseline	1.23 (0.7-2.02)	0% (0-0)	1.96 (1.76-2.31)	0% (0-0)
Congo	AM 25% Reduction	1.27 (0.72-2.11)	3.4% (2.08-4.58)	2.34 (2.07-2.79)	19.34% (17.87-20.67)
Congo	AM 50% Reduction	1.32 (0.73-2.2)	6.83% (4.14-9.27)	2.74 (2.4-3.3)	39.79% (36.4-42.92)
Congo	AM 75% Reduction	1.36 (0.75-2.3)	10.3% (6.07-14.09)	3.16 (2.73-3.86)	61.34% (55.38-66.9)
Congo	ITN 25% Reduction	1.23 (0.7-2.02)	0% (0-0.01)	1.96 (1.76-2.31)	0% (0-0.01)
Congo	ITN 50% Reduction	1.23 (0.7-2.02)	0.09% (0.05-0.16)	1.96 (1.76-2.31)	0.1% (0.05-0.16)
Congo	ITN 75% Reduction	1.23 (0.7-2.02)	0.03% (0.02-0.06)	1.96 (1.76-2.31)	0.03% (0.02-0.06)
Congo	Both 25% Reduction	1.27 (0.72-2.11)	3.4% (2.08-4.58)	2.34 (2.07-2.79)	19.34% (17.87-20.67)
Congo	Both 50% Reduction	1.32 (0.73-2.21)	6.93% (4.19-9.41)	2.74 (2.4-3.31)	39.91% (36.47-43.13)
Congo	Both 75% Reduction	1.36 (0.75-2.3)	10.34% (6.09-14.15)	3.16 (2.74-3.86)	61.39% (55.4-66.99)
Côte d'Ivoire	Baseline	8.29 (5.38-12.27)	0% (0-0)	9.3 (8.41-10.3)	0% (0-0)
Côte d'Ivoire	AM 25% Reduction	8.56 (5.48-12.84)	3.31% (1.83-4.68)	11.27 (10.04-12.65)	21.2% (19.43-22.83)
Côte d'Ivoire	AM 50% Reduction	8.84 (5.58-13.44)	6.64% (3.61-9.53)	13.35 (11.73-15.2)	43.58% (39.45-47.54)
Côte d'Ivoire	AM 75% Reduction	9.12 (5.67-14.06)	9.98% (5.33-14.58)	15.54 (13.46-17.94)	67.14% (60.02-74.22)
Côte d'Ivoire	ITN 25% Reduction	10.2 (5.93-17.68)	23.1% (10.28-44.11)	11.45 (9.28-14.84)	23.12% (10.29-44.11)
Côte d'Ivoire	ITN 50% Reduction	10.49 (6.02-18.27)	26.55% (11.81-48.9)	11.77 (9.4-15.34)	26.55% (11.82-48.89)

Name	Scenario	Cases (millions)	Increased cases (%)	Deaths (thousands)	Increased deaths (%)
Côte d'Ivoire	ITN 75% Reduction	10.71 (6.07-18.93)	29.24% (12.88-54.3)	12.02 (9.49-15.89)	29.25% (12.89-54.29)
Côte d'Ivoire	Both 25% Reduction	10.57 (6.05-18.43)	27.56% (12.4-50.19)	13.91 (11.09-18.15)	49.66% (31.87-76.24)
Côte d'Ivoire	Both 50% Reduction	11.05 (6.18-19.77)	33.28% (14.77-61.11)	16.68 (13-22.35)	79.46% (54.53-116.96)
Côte d'Ivoire	Both 75% Reduction	11.54 (6.3-21.19)	39.28% (17.12-72.72)	19.68 (14.97-27.04)	111.67% (77.96-162.56)
Democratic Republic of the Congo	Baseline	26.89 (19.6-35.91)	0% (0-0)	44.62 (32.2-62)	0% (0-0)
Democratic Republic of the Congo	AM 25% Reduction	27.65 (19.97-37.42)	2.84% (1.88-4.2)	57.13 (40.86-80.4)	28.05% (26.89-29.68)
Democratic Republic of the Congo	AM 50% Reduction	28.4 (20.32-38.93)	5.64% (3.68-8.4)	70.24 (49.78-100.09)	57.43% (54.6-61.43)
Democratic Republic of the Congo	AM 75% Reduction	29.14 (20.66-40.42)	8.38% (5.4-12.56)	83.92 (58.95-121.08)	88.1% (83.08-95.29)
Democratic Republic of the Congo	ITN 25% Reduction	28.31 (20.16-39.3)	5.3% (2.83-9.44)	46.97 (33.11-67.83)	5.27% (2.82-9.41)
Democratic Republic of the Congo	ITN 50% Reduction	28.5 (20.22-39.57)	6.01% (3.16-10.2)	47.28 (33.21-68.3)	5.98% (3.14-10.17)
Democratic Republic of the Congo	ITN 75% Reduction	28.59 (20.25-39.78)	6.33% (3.33-10.78)	47.43 (33.27-68.66)	6.31% (3.31-10.74)
Democratic Republic of the Congo	Both 25% Reduction	29.11 (20.53-40.82)	8.27% (4.76-13.67)	60.14 (42.01-87.7)	34.79% (30.48-41.45)
Democratic Republic of the Congo	Both 50% Reduction	29.93 (20.91-42.61)	11.33% (6.66-18.65)	74.01 (51.24-109.61)	65.9% (59.13-76.79)
Democratic Republic of the Congo	Both 75% Reduction	30.7 (21.22-44.21)	14.19% (8.28-23.12)	88.41 (60.6-132.51)	98.17% (88.19-113.72)
Equatorial Guinea	Baseline	0.35 (0.18-0.62)	0% (0-0)	0.66 (0.44-0.97)	0% (0-0)
Equatorial Guinea	AM 25% Reduction	0.36 (0.18-0.64)	1.15% (0-2.64)	0.72 (0.48-1.08)	9.94% (8.7-11.57)
Equatorial Guinea	AM 50% Reduction	0.36 (0.18-0.66)	2.27% (0-5.35)	0.79 (0.52-1.2)	20.07% (17.4-23.68)
Equatorial Guinea	AM 75% Reduction	0.36 (0.18-0.67)	3.38% (0-8.13)	0.86 (0.55-1.32)	30.36% (26.1-36.34)
Equatorial Guinea	ITN 25% Reduction	0.35 (0.18-0.62)	0.06% (0-0.15)	0.66 (0.44-0.97)	0.06% (0-0.15)
Equatorial Guinea	ITN 50% Reduction	0.35 (0.18-0.62)	0.1% (0-0.25)	0.66 (0.44-0.97)	0.1% (0-0.25)
Equatorial Guinea	ITN 75% Reduction	0.35 (0.18-0.63)	0.41% (0-1)	0.66 (0.44-0.98)	0.41% (0-1)
Equatorial Guinea	Both 25% Reduction	0.36 (0.18-0.64)	1.21% (0-2.81)	0.72 (0.48-1.08)	10.01% (8.7-11.75)
Equatorial Guinea	Both 50% Reduction	0.36 (0.18-0.66)	2.37% (0-5.64)	0.79 (0.52-1.2)	20.18% (17.4-24.01)
Equatorial Guinea	Both 75% Reduction	0.37 (0.18-0.68)	3.77% (0-9.28)	0.86 (0.55-1.34)	30.85% (26.1-37.8)
Eritrea	Baseline	0.1 (0.06-0.14)	0% (0-0)	0.2 (0.01-0.39)	0% (0-0)
Eritrea	AM 25% Reduction	0.1 (0.06-0.14)	1.97% (1.29-2.41)	0.21 (0.01-0.42)	6.09% (5.27-6.71)
Eritrea	AM 50% Reduction	0.1 (0.07-0.15)	3.98% (2.6-4.87)	0.22 (0.01-0.44)	12.38% (10.66-13.7)
Eritrea	AM 75% Reduction	0.11 (0.07-0.15)	6.01% (3.92-7.39)	0.23 (0.01-0.47)	18.87% (16.15-20.94)
Eritrea	ITN 25% Reduction	0.1 (0.06-0.14)	0.75% (0.39-1.21)	0.2 (0.01-0.39)	0.75% (0.39-1.21)
Eritrea	ITN 50% Reduction	0.1 (0.06-0.14)	0.78% (0.44-1.21)	0.2 (0.01-0.39)	0.78% (0.43-1.21)
Eritrea	ITN 75% Reduction	0.1 (0.06-0.14)	0.81% (0.45-1.26)	0.2 (0.01-0.39)	0.81% (0.45-1.26)
Eritrea	Both 25% Reduction	0.1 (0.07-0.14)	2.76% (1.74-3.59)	0.21 (0.01-0.42)	6.91% (5.74-7.92)

Name	Scenario	Cases (millions)	Increased cases (%)	Deaths (thousands)	Increased deaths (%)
Eritrea	Both 50% Reduction	0.1 (0.07-0.15)	4.78% (3.05-6.03)	0.22 (0.01-0.45)	13.24% (11.16-14.88)
Eritrea	Both 75% Reduction	0.11 (0.07-0.15)	6.86% (4.39-8.63)	0.23 (0.01-0.48)	19.81% (16.69-22.26)
Ethiopia	Baseline	2.36 (0.47-5.55)	0% (0-0)	4.76 (0.07-14.7)	0% (0-0)
Ethiopia	AM 25% Reduction	2.47 (0.49-5.83)	4.34% (3.64-5.05)	5.48 (0.08-17.06)	15.28% (14.49-16.03)
Ethiopia	AM 50% Reduction	2.57 (0.51-6.13)	8.83% (7.37-10.31)	6.26 (0.1-19.6)	31.66% (29.9-33.36)
Ethiopia	AM 75% Reduction	2.68 (0.53-6.43)	13.47% (11.18-15.81)	7.1 (0.11-22.36)	49.19% (46.21-52.09)
Ethiopia	ITN 25% Reduction	2.41 (0.48-5.7)	1.85% (1.33-2.72)	4.84 (0.07-15.1)	1.85% (1.33-2.72)
Ethiopia	ITN 50% Reduction	2.41 (0.48-5.71)	1.97% (1.41-2.84)	4.85 (0.08-15.12)	1.97% (1.41-2.84)
Ethiopia	ITN 75% Reduction	2.41 (0.48-5.72)	2.04% (1.46-2.95)	4.85 (0.08-15.13)	2.04% (1.46-2.94)
Ethiopia	Both 25% Reduction	2.51 (0.5-5.97)	6.29% (5.25-7.57)	5.59 (0.09-17.47)	17.43% (16.26-18.81)
Ethiopia	Both 50% Reduction	2.62 (0.52-6.27)	10.93% (9.08-12.87)	6.38 (0.1-20.06)	34.2% (32.06-36.48)
Ethiopia	Both 75% Reduction	2.73 (0.54-6.58)	15.71% (13.02-18.52)	7.24 (0.11-22.89)	52.13% (48.69-55.74)
Gabon	Baseline	0.53 (0.28-0.92)	0% (0-0)	0.53 (0.47-0.62)	0% (0-0)
Gabon	AM 25% Reduction	0.55 (0.28-0.99)	4.8% (1.9-6.89)	0.72 (0.62-0.86)	35.79% (32.08-38.41)
Gabon	AM 50% Reduction	0.58 (0.29-1.05)	9.69% (3.62-14.13)	0.92 (0.78-1.12)	74.56% (65-81.43)
Gabon	AM 75% Reduction	0.6 (0.29-1.12)	14.66% (5.16-21.71)	1.14 (0.93-1.42)	116.37% (98.58-129.38)
Gabon	ITN 25% Reduction	0.53 (0.28-0.93)	0.31% (0.08-0.44)	0.53 (0.47-0.62)	0.31% (0.08-0.44)
Gabon	ITN 50% Reduction	0.53 (0.28-0.92)	0.15% (0.06-0.21)	0.53 (0.47-0.62)	0.15% (0.06-0.21)
Gabon	ITN 75% Reduction	0.53 (0.28-0.93)	0.3% (0.11-0.42)	0.53 (0.47-0.62)	0.3% (0.11-0.42)
Gabon	Both 25% Reduction	0.55 (0.28-0.99)	5.12% (2.01-7.28)	0.72 (0.62-0.86)	36.2% (32.22-38.93)
Gabon	Both 50% Reduction	0.58 (0.29-1.05)	9.85% (3.67-14.32)	0.92 (0.78-1.13)	74.81% (65.07-81.74)
Gabon	Both 75% Reduction	0.6 (0.29-1.13)	14.97% (5.23-22.12)	1.15 (0.93-1.43)	116.96% (98.73-130.15)
Gambia	Baseline	0.15 (0.12-0.18)	0% (0-0)	0.69 (0.61-0.82)	0% (0-0)
Gambia	AM 25% Reduction	0.16 (0.13-0.2)	9.01% (6.84-10.15)	0.97 (0.85-1.17)	41.41% (38.56-42.88)
Gambia	AM 50% Reduction	0.18 (0.14-0.22)	18.7% (13.9-21.19)	1.3 (1.11-1.58)	89.24% (81.6-93.22)
Gambia	AM 75% Reduction	0.19 (0.14-0.25)	29.07% (21.17-33.3)	1.68 (1.4-2.07)	144.12% (129.22-152.12)
Gambia	ITN 25% Reduction	0.15 (0.12-0.19)	0.64% (0.38-0.92)	0.69 (0.61-0.83)	0.64% (0.38-0.92)
Gambia	ITN 50% Reduction	0.15 (0.12-0.19)	1.32% (0.81-1.87)	0.7 (0.61-0.84)	1.32% (0.81-1.87)
Gambia	ITN 75% Reduction	0.15 (0.12-0.19)	1.76% (1.08-2.49)	0.7 (0.62-0.84)	1.76% (1.09-2.5)
Gambia	Both 25% Reduction	0.17 (0.13-0.2)	9.71% (7.3-11.11)	0.98 (0.85-1.18)	42.31% (39.2-44.12)
Gambia	Both 50% Reduction	0.18 (0.14-0.23)	20.2% (14.86-23.32)	1.32 (1.12-1.61)	91.64% (83.13-96.58)
Gambia	Both 75% Reduction	0.2 (0.15-0.25)	31.21% (22.51-36.29)	1.71 (1.41-2.11)	148.18% (131.74-157.77)
Ghana	Baseline	6.68 (4.19-10.1)	0% (0-0)	11.07 (10.6-11.7)	0% (0-0)

Name	Scenario	Cases (millions)	Increased cases (%)	Deaths (thousands)	Increased deaths (%)
Ghana	AM 25% Reduction	7.07 (4.39-10.77)	5.8% (4.74-6.67)	15.3 (14.51-16.31)	38.24% (36.85-39.39)
Ghana	AM 50% Reduction	7.47 (4.59-11.48)	11.79% (9.54-13.63)	19.96 (18.73-21.45)	80.35% (76.71-83.34)
Ghana	AM 75% Reduction	7.88 (4.79-12.21)	17.96% (14.37-20.9)	25.07 (23.28-27.16)	126.48% (119.6-132.14)
Ghana	ITN 25% Reduction	6.71 (4.2-10.16)	0.43% (0.3-0.55)	11.12 (10.63-11.76)	0.43% (0.3-0.55)
Ghana	ITN 50% Reduction	6.75 (4.22-10.23)	1.02% (0.71-1.31)	11.18 (10.68-11.85)	1.02% (0.71-1.31)
Ghana	ITN 75% Reduction	6.77 (4.23-10.28)	1.39% (0.96-1.79)	11.22 (10.7-11.91)	1.39% (0.96-1.79)
Ghana	Both 25% Reduction	7.09 (4.4-10.83)	6.24% (5.11-7.18)	15.37 (14.56-16.39)	38.82% (37.34-40.05)
Ghana	Both 50% Reduction	7.54 (4.62-11.61)	12.86% (10.37-14.9)	20.16 (18.88-21.69)	82.08% (78.08-85.37)
Ghana	Both 75% Reduction	7.98 (4.83-12.39)	19.47% (15.48-22.69)	25.39 (23.5-27.56)	129.37% (121.74-135.55)
Guinea	Baseline	3.52 (2.06-5.63)	0% (0-0)	8.2 (6.88-10.1)	0% (0-0)
Guinea	AM 25% Reduction	3.59 (2.08-5.77)	1.88% (1.07-2.63)	9.01 (7.49-11.17)	9.78% (8.91-10.59)
Guinea	AM 50% Reduction	3.66 (2.1-5.92)	3.76% (2.1-5.31)	9.83 (8.12-12.29)	19.87% (17.95-21.65)
Guinea	AM 75% Reduction	3.72 (2.12-6.08)	5.65% (3.11-8.04)	10.68 (8.75-13.45)	30.25% (27.12-33.18)
Guinea	ITN 25% Reduction	3.54 (2.06-5.65)	0.33% (0.11-0.52)	8.23 (6.89-10.15)	0.33% (0.11-0.52)
Guinea	ITN 50% Reduction	3.54 (2.06-5.67)	0.48% (0.22-0.77)	8.24 (6.9-10.18)	0.48% (0.22-0.77)
Guinea	ITN 75% Reduction	3.54 (2.06-5.68)	0.56% (0.26-0.9)	8.25 (6.9-10.19)	0.56% (0.26-0.9)
Guinea	Both 25% Reduction	3.6 (2.08-5.8)	2.2% (1.22-3.09)	9.03 (7.5-11.22)	10.14% (9.07-11.09)
Guinea	Both 50% Reduction	3.67 (2.1-5.96)	4.24% (2.32-5.99)	9.88 (8.13-12.37)	20.42% (18.2-22.43)
Guinea	Both 75% Reduction	3.74 (2.12-6.12)	6.21% (3.36-8.85)	10.74 (8.77-13.55)	30.94% (27.42-34.17)
Guinea-Bissau	Baseline	0.23 (0.07-0.59)	0% (0-0)	0.68 (0.61-0.78)	0% (0-0)
Guinea-Bissau	AM 25% Reduction	0.25 (0.07-0.64)	6.95% (4.81-8.56)	0.88 (0.77-1.02)	29.24% (26.53-31.16)
Guinea-Bissau	AM 50% Reduction	0.26 (0.07-0.7)	14.29% (9.7-17.79)	1.1 (0.95-1.3)	61.92% (55.16-66.83)
Guinea-Bissau	AM 75% Reduction	0.28 (0.08-0.76)	22.01% (14.67-27.75)	1.35 (1.13-1.62)	98.29% (85.95-107.53)
Guinea-Bissau	ITN 25% Reduction	0.37 (0.09-1.11)	59.33% (28.99-87.21)	1.08 (0.79-1.46)	59.31% (28.98-87.24)
Guinea-Bissau	ITN 50% Reduction	0.37 (0.09-1.12)	60.16% (29.5-88.98)	1.09 (0.79-1.47)	60.18% (29.49-89.02)
Guinea-Bissau	ITN 75% Reduction	0.38 (0.09-1.14)	62.56% (30.6-92.77)	1.11 (0.8-1.5)	62.58% (30.58-92.81)
Guinea-Bissau	Both 25% Reduction	0.39 (0.09-1.19)	68.97% (34.49-100.73)	1.39 (0.99-1.89)	104.19% (62.41-142.69)
Guinea-Bissau	Both 50% Reduction	0.42 (0.09-1.29)	80.59% (40.72-117.25)	1.74 (1.21-2.4)	155.87% (99.13-207.91)
Guinea-Bissau	Both 75% Reduction	0.45 (0.1-1.41)	94.25% (47.82-137.91)	2.15 (1.46-3.02)	215.7% (139.86-286.8)
Kenya	Baseline	3.6 (2.02-6)	0% (0-0)	12.42 (11.8-13.2)	0% (0-0)
Kenya	AM 25% Reduction	3.87 (2.14-6.52)	7.52% (6.04-8.68)	16.86 (15.83-18.13)	35.81% (34.17-37.38)
Kenya	AM 50% Reduction	4.16 (2.27-7.07)	15.46% (12.31-17.94)	21.88 (20.3-23.81)	76.23% (72.02-80.37)
Kenya	AM 75% Reduction	4.46 (2.4-7.67)	23.82% (18.78-27.81)	27.52 (25.21-30.29)	121.62% (113.61-129.5)

Name	Scenario	Cases (millions)	Increased cases (%)	Deaths (thousands)	Increased deaths (%)
Kenya	ITN 25% Reduction	3.95 (2.15-6.8)	9.64% (6.67-13.43)	13.6 (12.58-14.95)	9.5% (6.6-13.26)
Kenya	ITN 50% Reduction	3.97 (2.16-6.85)	10.27% (7.31-14.24)	13.67 (12.65-15.06)	10.14% (7.25-14.06)
Kenya	ITN 75% Reduction	4 (2.17-6.91)	11.01% (7.83-15.28)	13.76 (12.71-15.19)	10.86% (7.75-15.1)
Kenya	Both 25% Reduction	4.24 (2.3-7.34)	17.73% (14.04-22.35)	18.45 (16.95-20.44)	48.6% (43.61-54.83)
Kenya	Both 50% Reduction	4.57 (2.44-7.98)	26.82% (20.95-33.05)	24.02 (21.85-26.87)	93.5% (85.14-103.58)
Kenya	Both 75% Reduction	4.92 (2.59-8.69)	36.58% (28.21-44.88)	30.35 (27.28-34.43)	144.41% (131.15-160.81)
Liberia	Baseline	1.74 (1.18-2.45)	0% (0-0)	2.01 (1.73-2.42)	0% (0-0)
Liberia	AM 25% Reduction	1.78 (1.19-2.55)	2.16% (0.53-4.04)	2.66 (2.26-3.27)	32.74% (30.63-35.17)
Liberia	AM 50% Reduction	1.82 (1.19-2.64)	4.2% (0.92-7.92)	3.34 (2.79-4.17)	66.58% (61.34-72.51)
Liberia	AM 75% Reduction	1.85 (1.2-2.73)	6.13% (1.18-11.66)	4.04 (3.32-5.13)	101.43% (92.04-111.9)
Liberia	ITN 25% Reduction	1.75 (1.18-2.48)	0.59% (0.11-1.2)	2.02 (1.73-2.45)	0.59% (0.11-1.2)
Liberia	ITN 50% Reduction	1.76 (1.19-2.5)	1.12% (0.26-2.17)	2.03 (1.73-2.47)	1.12% (0.26-2.17)
Liberia	ITN 75% Reduction	1.77 (1.19-2.52)	1.62% (0.34-3.12)	2.04 (1.74-2.5)	1.62% (0.34-3.12)
Liberia	Both 25% Reduction	1.79 (1.19-2.57)	2.75% (0.64-5.15)	2.68 (2.26-3.31)	33.51% (30.77-36.62)
Liberia	Both 50% Reduction	1.83 (1.19-2.69)	5.22% (1.05-9.87)	3.37 (2.79-4.25)	68.21% (61.55-75.63)
Liberia	Both 75% Reduction	1.87 (1.2-2.8)	7.53% (1.31-14.46)	4.09 (3.33-5.26)	104.09% (92.29-117.22)
Madagascar	Baseline	2.16 (1.62-2.78)	0% (0-0)	5.35 (0.19-10.1)	0% (0-0)
Madagascar	AM 25% Reduction	2.29 (1.7-2.95)	5.78% (5.13-6.48)	6.64 (0.23-12.61)	24.07% (23.17-24.88)
Madagascar	AM 50% Reduction	2.42 (1.79-3.14)	11.79% (10.42-13.31)	8.05 (0.28-15.4)	50.46% (48.33-52.46)
Madagascar	AM 75% Reduction	2.55 (1.87-3.34)	18.05% (15.87-20.48)	9.59 (0.33-18.47)	79.29% (75.59-82.92)
Madagascar	ITN 25% Reduction	2.17 (1.62-2.78)	0.07% (0.05-0.09)	5.35 (0.19-10.11)	0.07% (0.05-0.09)
Madagascar	ITN 50% Reduction	2.17 (1.62-2.79)	0.32% (0.22-0.4)	5.37 (0.19-10.14)	0.32% (0.22-0.4)
Madagascar	ITN 75% Reduction	2.18 (1.63-2.8)	0.84% (0.58-1.07)	5.39 (0.19-10.21)	0.83% (0.58-1.06)
Madagascar	Both 25% Reduction	2.29 (1.7-2.96)	5.85% (5.2-6.56)	6.64 (0.23-12.62)	24.16% (23.23-24.96)
Madagascar	Both 50% Reduction	2.43 (1.79-3.15)	12.13% (10.74-13.67)	8.07 (0.28-15.45)	50.91% (48.68-52.92)
Madagascar	Both 75% Reduction	2.57 (1.89-3.37)	18.99% (16.68-21.52)	9.67 (0.34-18.63)	80.71% (76.66-84.46)
Malawi	Baseline	3.88 (2.68-5.47)	0% (0-0)	6.48 (5.78-7.46)	0% (0-0)
Malawi	AM 25% Reduction	4.06 (2.78-5.77)	4.81% (3.86-5.56)	8.31 (7.35-9.64)	28.27% (27.12-29.19)
Malawi	AM 50% Reduction	4.25 (2.89-6.09)	9.74% (7.76-11.35)	10.29 (9.02-12.03)	58.87% (56.01-61.22)
Malawi	AM 75% Reduction	4.45 (2.99-6.41)	14.78% (11.64-17.23)	12.43 (10.79-14.62)	91.87% (86.62-96)
Malawi	ITN 25% Reduction	3.91 (2.69-5.53)	0.84% (0.58-1.13)	6.53 (5.81-7.54)	0.84% (0.58-1.13)
Malawi	ITN 50% Reduction	3.94 (2.71-5.59)	1.57% (1.11-2.09)	6.58 (5.84-7.62)	1.57% (1.11-2.09)
Malawi	ITN 75% Reduction	3.96 (2.72-5.63)	2.15% (1.52-2.88)	6.62 (5.87-7.67)	2.15% (1.52-2.88)

Name	Scenario	Cases (millions)	Increased cases (%)	Deaths (thousands)	Increased deaths (%)
Malawi	Both 25% Reduction	4.1 (2.8-5.83)	5.68% (4.54-6.53)	8.38 (7.39-9.73)	29.34% (27.94-30.37)
Malawi	Both 50% Reduction	4.32 (2.92-6.2)	11.38% (8.96-13.23)	10.45 (9.12-12.23)	61.25% (57.73-63.92)
Malawi	Both 75% Reduction	4.54 (3.03-6.56)	17.08% (13.24-19.91)	12.68 (10.94-14.95)	95.72% (89.29-100.43)
Mali	Baseline	7.38 (5.2-10.48)	0% (0-0)	11.85 (10.1-13.8)	0% (0-0)
Mali	AM 25% Reduction	7.57 (5.3-10.8)	2.55% (1.99-3.06)	13.04 (11.06-15.27)	10.08% (9.47-10.62)
Mali	AM 50% Reduction	7.76 (5.41-11.13)	5.15% (3.99-6.19)	14.29 (12.04-16.8)	20.58% (19.26-21.77)
Mali	AM 75% Reduction	7.95 (5.51-11.46)	7.79% (6.02-9.4)	15.58 (13.07-18.42)	31.51% (29.36-33.47)
Mali	ITN 25% Reduction	7.82 (5.39-11.34)	5.96% (3.65-8.22)	12.55 (10.47-14.93)	5.96% (3.65-8.22)
Mali	ITN 50% Reduction	7.85 (5.4-11.4)	6.32% (3.89-8.76)	12.6 (10.49-15.01)	6.32% (3.89-8.77)
Mali	ITN 75% Reduction	7.88 (5.42-11.46)	6.75% (4.15-9.37)	12.65 (10.52-15.09)	6.75% (4.15-9.37)
Mali	Both 25% Reduction	8.01 (5.51-11.66)	8.58% (5.93-11.29)	13.81 (11.48-16.48)	16.55% (13.7-19.45)
Mali	Both 50% Reduction	8.24 (5.64-12.07)	11.67% (8.38-15.13)	15.17 (12.55-18.22)	28.05% (24.28-32)
Mali	Both 75% Reduction	8.48 (5.77-12.47)	14.86% (10.87-19.02)	16.6 (13.66-20.04)	40.13% (35.26-45.18)
Mauritania	Baseline	0.17 (0.08-0.3)	0% (0-0)	1.4 (1.19-1.8)	0% (0-0)
Mauritania	AM 25% Reduction	0.18 (0.08-0.32)	5.13% (3.8-6)	1.67 (1.4-2.17)	19.44% (17.42-20.56)
Mauritania	AM 50% Reduction	0.19 (0.09-0.33)	10.46% (7.71-12.33)	1.96 (1.62-2.58)	40.55% (35.97-43.18)
Mauritania	AM 75% Reduction	0.2 (0.09-0.35)	16% (11.76-18.99)	2.28 (1.85-3.02)	63.41% (55.67-68.02)
Mauritania	ITN 25% Reduction	0.19 (0.09-0.34)	9.79% (5.45-15.25)	1.53 (1.25-2.07)	9.73% (5.39-15.26)
Mauritania	ITN 50% Reduction	0.19 (0.09-0.34)	10.08% (6.04-15.64)	1.54 (1.26-2.08)	10.04% (5.97-15.65)
Mauritania	ITN 75% Reduction	0.19 (0.09-0.34)	10.11% (6.07-15.69)	1.54 (1.26-2.08)	10.08% (5.99-15.71)
Mauritania	Both 25% Reduction	0.2 (0.09-0.36)	15.35% (9.88-21.95)	1.83 (1.48-2.5)	31.03% (24.19-38.96)
Mauritania	Both 50% Reduction	0.21 (0.09-0.39)	21.34% (14.1-29.29)	2.16 (1.71-2.97)	54.38% (43.87-64.87)
Mauritania	Both 75% Reduction	0.22 (0.1-0.41)	27.33% (18.27-36.31)	2.51 (1.96-3.47)	79.37% (64.88-92.65)
Mozambique	Baseline	9.01 (7.16-11.16)	0% (0-0)	14.43 (11.9-18.4)	0% (0-0)
Mozambique	AM 25% Reduction	9.35 (7.36-11.67)	3.77% (2.85-4.58)	17.98 (14.7-23.11)	24.62% (23.51-25.6)
Mozambique	AM 50% Reduction	9.69 (7.56-12.2)	7.57% (5.64-9.3)	21.75 (17.62-28.2)	50.79% (48.08-53.24)
Mozambique	AM 75% Reduction	10.03 (7.76-12.74)	11.37% (8.34-14.18)	25.75 (20.67-33.68)	78.5% (73.66-83.02)
Mozambique	ITN 25% Reduction	10.11 (7.71-13.09)	12.25% (7.65-17.32)	16.19 (12.81-21.59)	12.25% (7.65-17.31)
Mozambique	ITN 50% Reduction	10.19 (7.75-13.2)	13.15% (8.31-18.27)	16.32 (12.89-21.76)	13.14% (8.31-18.26)
Mozambique	ITN 75% Reduction	10.24 (7.78-13.29)	13.72% (8.67-19.07)	16.4 (12.93-21.91)	13.72% (8.66-19.07)
Mozambique	Both 25% Reduction	10.47 (7.92-13.62)	16.22% (10.67-22.07)	20.13 (15.82-26.97)	39.57% (32.9-46.6)
Mozambique	Both 50% Reduction	10.86 (8.14-14.32)	20.6% (13.66-28.28)	24.39 (18.96-33.08)	69.06% (59.34-79.81)
Mozambique	Both 75% Reduction	11.24 (8.33-14.99)	24.83% (16.35-34.31)	28.86 (22.19-39.6)	100.06% (86.5-115.22)

Name	Scenario	Cases (millions)	Increased cases (%)	Deaths (thousands)	Increased deaths (%)
Namibia	Baseline	0.05 (0.04-0.06)	0% (0-0)	0.13 (0-0.24)	0% (0-0)
Namibia	AM 25% Reduction	0.06 (0.04-0.07)	7.44% (5.19-9.7)	0.2 (0.01-0.36)	47.82% (44.74-50.93)
Namibia	AM 50% Reduction	0.06 (0.05-0.08)	15.22% (10.39-20.1)	0.27 (0.01-0.5)	101.84% (93.39-110.38)
Namibia	AM 75% Reduction	0.06 (0.05-0.08)	23.34% (15.7-31.18)	0.35 (0.01-0.67)	162.44% (146.18-179.09)
Namibia	ITN 25% Reduction	0.05 (0.04-0.06)	0% (0-0)	0.13 (0-0.24)	0% (0-0)
Namibia	ITN 50% Reduction	0.05 (0.04-0.06)	0% (0-0)	0.13 (0-0.24)	0% (0-0)
Namibia	ITN 75% Reduction	0.05 (0.04-0.06)	0% (0-0)	0.13 (0-0.24)	0% (0-0)
Namibia	Both 25% Reduction	0.06 (0.04-0.07)	7.44% (5.19-9.7)	0.2 (0.01-0.36)	47.82% (44.74-50.93)
Namibia	Both 50% Reduction	0.06 (0.05-0.08)	15.22% (10.39-20.1)	0.27 (0.01-0.5)	101.84% (93.39-110.38)
Namibia	Both 75% Reduction	0.06 (0.05-0.08)	23.34% (15.7-31.18)	0.35 (0.01-0.67)	162.44% (146.18-179.09)
Niger	Baseline	8 (4.22-13.36)	0% (0-0)	17.08 (12.3-24.2)	0% (0-0)
Niger	AM 25% Reduction	8.17 (4.26-13.75)	2.09% (1.18-2.9)	18.92 (13.5-27.01)	10.73% (9.74-11.6)
Niger	AM 50% Reduction	8.34 (4.31-14.14)	4.19% (2.33-5.87)	20.81 (14.72-29.95)	21.82% (19.64-23.77)
Niger	AM 75% Reduction	8.51 (4.36-14.55)	6.31% (3.45-8.91)	22.77 (15.95-33.04)	33.28% (29.7-36.53)
Niger	ITN 25% Reduction	8.12 (4.25-13.66)	1.48% (0.78-2.25)	17.34 (12.4-24.75)	1.48% (0.78-2.25)
Niger	ITN 50% Reduction	8.13 (4.25-13.69)	1.6% (0.84-2.44)	17.36 (12.4-24.79)	1.6% (0.84-2.44)
Niger	ITN 75% Reduction	8.14 (4.25-13.71)	1.72% (0.91-2.63)	17.38 (12.41-24.84)	1.72% (0.91-2.63)
Niger	Both 25% Reduction	8.29 (4.3-14.06)	3.58% (2.03-5.24)	19.19 (13.61-27.62)	12.34% (10.66-14.14)
Niger	Both 50% Reduction	8.47 (4.35-14.5)	5.8% (3.22-8.5)	21.13 (14.84-30.7)	23.7% (20.67-26.85)
Niger	Both 75% Reduction	8.65 (4.4-14.94)	8.05% (4.38-11.8)	23.14 (16.1-33.92)	35.47% (30.86-40.16)
Nigeria	Baseline	57.18 (38.94-81.23)	0% (0-0)	95.84 (80.8-117)	0% (0-0)
Nigeria	AM 25% Reduction	59.69 (40.29-85.57)	4.38% (3.46-5.34)	123.54 (103.05-152.24)	28.9% (27.54-30.12)
Nigeria	AM 50% Reduction	62.23 (41.61-90.06)	8.82% (6.86-10.87)	153.37 (126.57-190.85)	60.02% (56.65-63.12)
Nigeria	AM 75% Reduction	64.8 (42.9-94.67)	13.31% (10.16-16.54)	185.34 (151.31-233.13)	93.38% (87.27-99.25)
Nigeria	ITN 25% Reduction	59.32 (39.91-85.61)	3.74% (2.5-5.39)	99.52 (82.87-123.48)	3.84% (2.57-5.53)
Nigeria	ITN 50% Reduction	59.46 (39.98-85.78)	3.98% (2.66-5.6)	99.76 (83.01-123.72)	4.09% (2.73-5.74)
Nigeria	ITN 75% Reduction	59.56 (40.02-85.98)	4.16% (2.77-5.84)	99.93 (83.1-124.01)	4.27% (2.85-6)
Nigeria	Both 25% Reduction	61.91 (41.26-89.93)	8.27% (5.96-10.71)	128.23 (105.7-160.08)	33.79% (30.82-36.82)
Nigeria	Both 50% Reduction	64.56 (42.59-94.64)	12.89% (9.38-16.51)	159.17 (129.63-200.86)	66.07% (60.44-71.67)
Nigeria	Both 75% Reduction	67.24 (43.89-99.64)	17.59% (12.72-22.67)	192.36 (154.75-245.31)	100.7% (91.52-109.66)
Rwanda	Baseline	5.98 (4.37-7.68)	0% (0-0)	3.24 (3.02-3.63)	0% (0-0)
Rwanda	AM 25% Reduction	6.3 (4.54-8.16)	5.28% (3.95-6.31)	3.98 (3.66-4.49)	22.63% (21.08-23.82)
Rwanda	AM 50% Reduction	6.63 (4.72-8.67)	10.74% (7.95-12.91)	4.78 (4.33-5.45)	47.22% (43.52-50.12)

Name	Scenario	Cases (millions)	Increased cases (%)	Deaths (thousands)	Increased deaths (%)
Rwanda	AM 75% Reduction	6.96 (4.89-9.2)	16.35% (11.98-19.78)	5.64 (5.05-6.5)	73.86% (67.33-79.03)
Rwanda	ITN 25% Reduction	6.77 (4.76-9.11)	13.1% (9.05-18.65)	3.67 (3.29-4.31)	13.09% (9.05-18.65)
Rwanda	ITN 50% Reduction	6.88 (4.81-9.28)	14.97% (10.2-20.83)	3.73 (3.33-4.39)	14.97% (10.19-20.83)
Rwanda	ITN 75% Reduction	6.95 (4.85-9.4)	16.12% (10.96-22.44)	3.77 (3.35-4.44)	16.11% (10.95-22.43)
Rwanda	Both 25% Reduction	7.13 (4.95-9.61)	19.09% (13.39-25.22)	4.5 (3.99-5.29)	38.71% (32.07-45.85)
Rwanda	Both 50% Reduction	7.58 (5.19-10.34)	26.6% (18.89-34.63)	5.46 (4.77-6.5)	68.31% (58.07-78.99)
Rwanda	Both 75% Reduction	8.01 (5.43-11.09)	33.9% (24.21-44.47)	6.49 (5.61-7.84)	100.08% (85.61-115.87)
Senegal	Baseline	0.88 (0.62-1.16)	0% (0-0)	4.48 (4.26-4.78)	0% (0-0)
Senegal	AM 25% Reduction	0.94 (0.65-1.24)	5.79% (4.81-6.58)	5.54 (5.22-5.95)	23.59% (22.45-24.51)
Senegal	AM 50% Reduction	0.99 (0.68-1.32)	11.8% (9.74-13.53)	6.7 (6.25-7.25)	49.45% (46.67-51.75)
Senegal	AM 75% Reduction	1.04 (0.71-1.41)	18.05% (14.79-20.89)	7.96 (7.36-8.7)	77.67% (72.74-81.93)
Senegal	ITN 25% Reduction	0.89 (0.62-1.17)	0.47% (0.29-0.62)	4.5 (4.27-4.81)	0.47% (0.29-0.62)
Senegal	ITN 50% Reduction	0.89 (0.62-1.18)	0.8% (0.51-1.05)	4.52 (4.28-4.83)	0.8% (0.51-1.05)
Senegal	ITN 75% Reduction	0.9 (0.62-1.18)	1.31% (0.84-1.7)	4.54 (4.3-4.86)	1.31% (0.84-1.7)
Senegal	Both 25% Reduction	0.94 (0.65-1.25)	6.28% (5.2-7.24)	5.56 (5.24-5.99)	24.17% (22.9-25.28)
Senegal	Both 50% Reduction	1 (0.68-1.33)	12.66% (10.4-14.7)	6.75 (6.29-7.33)	50.59% (47.56-53.31)
Senegal	Both 75% Reduction	1.06 (0.72-1.43)	19.51% (15.9-22.89)	8.06 (7.43-8.84)	79.86% (74.41-84.94)
Sierra Leone	Baseline	2.45 (1.43-3.98)	0% (0-0)	6.56 (5.52-7.77)	0% (0-0)
Sierra Leone	AM 25% Reduction	2.53 (1.45-4.16)	3.1% (1.38-4.52)	7.99 (6.61-9.59)	21.78% (19.73-23.42)
Sierra Leone	AM 50% Reduction	2.6 (1.47-4.35)	6.21% (2.68-9.22)	9.5 (7.72-11.56)	44.68% (39.82-48.73)
Sierra Leone	AM 75% Reduction	2.68 (1.49-4.54)	9.31% (3.87-14.09)	11.07 (8.84-13.68)	68.7% (60.22-76.02)
Sierra Leone	ITN 25% Reduction	2.88 (1.5-5.36)	17.66% (4.36-34.7)	7.72 (5.76-10.47)	17.68% (4.37-34.7)
Sierra Leone	ITN 50% Reduction	2.93 (1.52-5.49)	19.55% (5.97-38.07)	7.85 (5.85-10.73)	19.56% (5.98-38.08)
Sierra Leone	ITN 75% Reduction	2.97 (1.52-5.63)	20.99% (6.32-41.42)	7.94 (5.87-10.99)	21% (6.33-41.43)
Sierra Leone	Both 25% Reduction	2.97 (1.53-5.6)	20.99% (6.48-40.8)	9.38 (6.94-12.93)	42.91% (25.74-66.38)
Sierra Leone	Both 50% Reduction	3.07 (1.54-5.97)	25.27% (7.58-50.02)	11.2 (8.09-15.9)	70.65% (46.49-104.59)
Sierra Leone	Both 75% Reduction	3.17 (1.55-6.32)	29.34% (8.45-58.81)	13.1 (9.23-19.06)	99.61% (67.28-145.33)
Somalia	Baseline	0.51 (0.31-0.77)	0% (0-0)	1.32 (0.04-2.96)	0% (0-0)
Somalia	AM 25% Reduction	0.53 (0.31-0.79)	2.33% (1.85-2.68)	1.42 (0.04-3.2)	7.56% (7.02-7.96)
Somalia	AM 50% Reduction	0.54 (0.32-0.81)	4.71% (3.73-5.42)	1.52 (0.04-3.44)	15.4% (14.26-16.27)
Somalia	AM 75% Reduction	0.55 (0.32-0.84)	7.12% (5.63-8.22)	1.63 (0.04-3.7)	23.54% (21.72-24.92)
Somalia	ITN 25% Reduction	0.52 (0.31-0.79)	1.3% (0.91-1.8)	1.33 (0.04-3.01)	1.3% (0.91-1.8)
Somalia	ITN 50% Reduction	0.52 (0.31-0.79)	1.38% (0.95-1.88)	1.33 (0.04-3.02)	1.38% (0.95-1.88)

Name	Scenario	Cases (millions)	Increased cases (%)	Deaths (thousands)	Increased deaths (%)
Somalia	ITN 75% Reduction	0.52 (0.31-0.79)	1.37% (0.94-1.86)	1.33 (0.04-3.02)	1.37% (0.94-1.86)
Somalia	Both 25% Reduction	0.53 (0.31-0.81)	3.67% (2.84-4.33)	1.43 (0.04-3.25)	8.96% (8.09-9.67)
Somalia	Both 50% Reduction	0.55 (0.32-0.83)	6.13% (4.76-7.09)	1.54 (0.04-3.49)	16.97% (15.42-18.06)
Somalia	Both 75% Reduction	0.56 (0.33-0.85)	8.56% (6.63-9.86)	1.65 (0.04-3.75)	25.19% (22.9-26.79)
South Africa	Baseline	0.01 (0.01-0.01)	0% (0-0)	0.07 (0.07-0.07)	0% (0-0)
South Africa	AM 25% Reduction	0.01 (0.01-0.01)	6.6% (4.57-9.16)	0.1 (0.09-0.1)	40.32% (37.6-43.7)
South Africa	AM 50% Reduction	0.01 (0.01-0.01)	13.46% (9.19-18.97)	0.13 (0.12-0.13)	85.25% (78.22-94.34)
South Africa	AM 75% Reduction	0.01 (0.01-0.01)	20.58% (13.83-29.46)	0.16 (0.15-0.17)	135.04% (121.67-152.52)
South Africa	ITN 25% Reduction	0.01 (0.01-0.01)	0.01% (0-0.02)	0.07 (0.07-0.07)	0.01% (0-0.02)
South Africa	ITN 50% Reduction	0.01 (0.01-0.01)	0.01% (0-0.02)	0.07 (0.07-0.07)	0.01% (0-0.03)
South Africa	ITN 75% Reduction	0.01 (0.01-0.01)	0.01% (0-0.03)	0.07 (0.07-0.07)	0.01% (0-0.03)
South Africa	Both 25% Reduction	0.01 (0.01-0.01)	6.61% (4.58-9.18)	0.1 (0.09-0.1)	40.34% (37.61-43.75)
South Africa	Both 50% Reduction	0.01 (0.01-0.01)	13.47% (9.2-19)	0.13 (0.12-0.13)	85.27% (78.22-94.4)
South Africa	Both 75% Reduction	0.01 (0.01-0.01)	20.59% (13.84-29.49)	0.16 (0.15-0.17)	135.06% (121.68-152.56)
South Sudan	Baseline	2.59 (1.58-4.05)	0% (0-0)	5.36 (4.08-7.49)	0% (0-0)
South Sudan	AM 25% Reduction	2.6 (1.58-4.07)	0.5% (0.3-0.66)	5.46 (4.15-7.65)	1.95% (1.75-2.12)
South Sudan	AM 50% Reduction	2.62 (1.59-4.1)	1% (0.6-1.33)	5.57 (4.22-7.81)	3.92% (3.51-4.26)
South Sudan	AM 75% Reduction	2.63 (1.59-4.13)	1.51% (0.9-2)	5.67 (4.3-7.97)	5.9% (5.27-6.42)
South Sudan	ITN 25% Reduction	3.15 (1.75-5.56)	21.78% (10.7-37.39)	6.52 (4.52-10.29)	21.78% (10.7-37.39)
South Sudan	ITN 50% Reduction	3.18 (1.75-5.61)	22.86% (11.03-38.51)	6.58 (4.53-10.38)	22.87% (11.03-38.52)
South Sudan	ITN 75% Reduction	3.2 (1.76-5.66)	23.64% (11.41-39.82)	6.62 (4.55-10.47)	23.64% (11.41-39.83)
South Sudan	Both 25% Reduction	3.18 (1.75-5.59)	22.72% (11.03-38.13)	6.67 (4.6-10.5)	24.49% (12.63-40.13)
South Sudan	Both 50% Reduction	3.21 (1.76-5.67)	23.91% (11.67-40)	6.83 (4.69-10.79)	27.49% (14.9-44.05)
South Sudan	Both 75% Reduction	3.24 (1.77-5.75)	25.22% (12.31-42.06)	7 (4.78-11.1)	30.64% (17.18-48.22)
Sudan	Baseline	1.95 (0.9-3.69)	0% (0-0)	5 (0.12-12.3)	0% (0-0)
Sudan	AM 25% Reduction	2.06 (0.95-3.92)	5.65% (4.68-6.31)	6.11 (0.15-15.11)	22.12% (20.93-22.88)
Sudan	AM 50% Reduction	2.18 (0.99-4.16)	11.57% (9.51-12.95)	7.32 (0.17-18.23)	46.33% (43.52-48.19)
Sudan	AM 75% Reduction	2.3 (1.03-4.42)	17.74% (14.49-19.94)	8.64 (0.2-21.66)	72.78% (67.87-76.08)
Sudan	ITN 25% Reduction	1.96 (0.91-3.71)	0.53% (0.32-0.77)	5.03 (0.12-12.39)	0.53% (0.32-0.77)
Sudan	ITN 50% Reduction	1.97 (0.91-3.72)	0.7% (0.41-1)	5.04 (0.12-12.42)	0.7% (0.41-0.99)
Sudan	ITN 75% Reduction	1.97 (0.91-3.73)	0.79% (0.46-1.12)	5.04 (0.12-12.44)	0.79% (0.46-1.12)
Sudan	Both 25% Reduction	2.08 (0.95-3.94)	6.22% (5.04-6.96)	6.14 (0.15-15.21)	22.77% (21.33-23.63)
Sudan	Both 50% Reduction	2.2 (0.99-4.2)	12.32% (10.01-13.81)	7.37 (0.17-18.36)	47.33% (44.13-49.3)

Name	Scenario	Cases (millions)	Increased cases (%)	Deaths (thousands)	Increased deaths (%)
Sudan	Both 75% Reduction	2.32 (1.04-4.46)	18.62% (15.09-20.97)	8.71 (0.2-21.84)	74.08% (68.67-77.53)
Tanzania	Baseline	7 (4.68-10.09)	0% (0-0)	21.55 (20.1-23.5)	0% (0-0)
Tanzania	AM 25% Reduction	7.46 (4.96-10.82)	6.65% (6-7.27)	29.3 (27.16-32.14)	35.97% (35.14-36.76)
Tanzania	AM 50% Reduction	7.95 (5.25-11.59)	13.6% (12.19-14.91)	37.94 (34.95-41.85)	76.04% (73.88-78.1)
Tanzania	AM 75% Reduction	8.45 (5.55-12.4)	20.82% (18.57-22.92)	47.51 (43.49-52.71)	120.46% (116.36-124.31)
Tanzania	ITN 25% Reduction	7.22 (4.78-10.5)	3.16% (2.26-4.03)	22.23 (20.55-24.45)	3.15% (2.26-4.03)
Tanzania	ITN 50% Reduction	7.31 (4.83-10.66)	4.41% (3.21-5.62)	22.5 (20.75-24.82)	4.4% (3.21-5.62)
Tanzania	ITN 75% Reduction	7.42 (4.88-10.86)	5.98% (4.37-7.63)	22.84 (20.98-25.29)	5.98% (4.37-7.63)
Tanzania	Both 25% Reduction	7.69 (5.08-11.21)	9.95% (8.65-11.11)	30.21 (27.85-33.29)	40.17% (38.54-41.64)
Tanzania	Both 50% Reduction	8.28 (5.43-12.17)	18.37% (16.04-20.58)	39.53 (36.15-43.91)	83.44% (79.84-86.85)
Tanzania	Both 75% Reduction	8.92 (5.79-13.21)	27.54% (23.89-30.89)	50.15 (45.44-56.13)	132.71% (126.07-138.84)
Togo	Baseline	2.11 (1.51-2.9)	0% (0-0)	5.13 (4.17-6.41)	0% (0-0)
Togo	AM 25% Reduction	2.21 (1.57-3.06)	4.68% (3.82-5.49)	6.41 (5.16-8.06)	24.83% (23.8-25.79)
Togo	AM 50% Reduction	2.31 (1.62-3.23)	9.49% (7.71-11.17)	7.78 (6.22-9.87)	51.63% (49.16-53.96)
Togo	AM 75% Reduction	2.41 (1.68-3.39)	14.43% (11.65-17.02)	9.26 (7.34-11.83)	80.48% (76.11-84.59)
Togo	ITN 25% Reduction	2.71 (1.79-4.04)	28.58% (19.02-39.11)	6.6 (4.96-8.92)	28.59% (19.02-39.11)
Togo	ITN 50% Reduction	2.72 (1.8-4.05)	29.06% (19.52-39.73)	6.62 (4.98-8.96)	29.06% (19.52-39.73)
Togo	ITN 75% Reduction	2.72 (1.8-4.05)	28.93% (19.43-39.54)	6.62 (4.98-8.95)	28.93% (19.44-39.55)
Togo	Both 25% Reduction	2.83 (1.86-4.22)	33.98% (23.57-45.34)	8.2 (6.14-11.11)	59.77% (47.35-73.31)
Togo	Both 50% Reduction	2.95 (1.93-4.42)	39.94% (28.15-52.31)	9.95 (7.4-13.52)	93.8% (77.47-110.93)
Togo	Both 75% Reduction	3.07 (1.99-4.59)	45.39% (32.29-58.24)	11.77 (8.7-16)	129.33% (108.66-149.59)
Uganda	Baseline	12.36 (7.62-18.97)	0% (0-0)	13.2 (11.7-15.2)	0% (0-0)
Uganda	AM 25% Reduction	13.23 (8.07-20.53)	7.07% (5.88-8.22)	19.74 (17.3-22.96)	49.48% (47.83-51.09)
Uganda	AM 50% Reduction	14.14 (8.52-22.17)	14.43% (11.82-16.88)	27.08 (23.45-31.84)	105.09% (100.44-109.47)
Uganda	AM 75% Reduction	15.08 (8.98-23.92)	22.07% (17.84-26.08)	35.27 (30.18-41.93)	167.14% (157.91-175.87)
Uganda	ITN 25% Reduction	15.35 (8.79-25.27)	24.25% (15.29-33.22)	16.4 (13.49-20.25)	24.24% (15.29-33.22)
Uganda	ITN 50% Reduction	15.68 (8.94-25.87)	26.93% (17.26-36.35)	16.76 (13.72-20.73)	26.93% (17.26-36.35)
Uganda	ITN 75% Reduction	15.89 (9.01-26.29)	28.56% (18.23-38.61)	16.97 (13.83-21.07)	28.56% (18.24-38.6)
Uganda	Both 25% Reduction	16.38 (9.32-27.09)	32.54% (22.26-42.81)	24.43 (19.97-30.3)	85.05% (70.7-99.34)
Uganda	Both 50% Reduction	17.68 (9.9-29.51)	43.04% (29.82-55.58)	33.85 (27.23-42.39)	156.37% (132.72-178.85)
Uganda	Both 75% Reduction	18.92 (10.42-32)	53.15% (36.71-68.69)	44.25 (35.01-56.11)	235.14% (199.24-269.11)
Zambia	Baseline	2.72 (1.71-4.1)	0% (0-0)	7.52 (6.89-8.39)	0% (0-0)
Zambia	AM 25% Reduction	2.89 (1.8-4.42)	6.47% (5.29-7.82)	10.9 (9.88-12.32)	45% (43.41-46.84)

Name	Scenario	Cases (millions)	Increased cases (%)	Deaths (thousands)	Increased deaths (%)
Zambia	AM 50% Reduction	3.08 (1.89-4.75)	13.17% (10.66-16.09)	14.67 (13.14-16.79)	95.1% (90.78-100.11)
Zambia	AM 75% Reduction	3.27 (1.98-5.11)	20.11% (16.05-24.8)	18.84 (16.68-21.84)	150.53% (142.09-160.3)
Zambia	ITN 25% Reduction	3.15 (1.89-5.06)	16.02% (10.31-23.65)	8.72 (7.6-10.37)	16.02% (10.31-23.66)
Zambia	ITN 50% Reduction	3.21 (1.91-5.17)	18.1% (11.77-26.27)	8.88 (7.7-10.59)	18.1% (11.77-26.27)
Zambia	ITN 75% Reduction	3.26 (1.93-5.27)	19.74% (12.85-28.66)	9 (7.78-10.79)	19.74% (12.85-28.66)
Zambia	Both 25% Reduction	3.35 (1.99-5.43)	23.22% (16.21-32.53)	12.62 (10.91-15.14)	67.82% (58.28-80.51)
Zambia	Both 50% Reduction	3.6 (2.1-5.92)	32.27% (23.15-44.53)	17.15 (14.63-20.9)	128.02% (112.28-149.16)
Zambia	Both 75% Reduction	3.85 (2.22-6.43)	41.46% (29.83-56.91)	22.19 (18.66-27.46)	195.07% (170.81-227.31)
Zimbabwe	Baseline	0.58 (0.39-0.79)	0% (0-0)	1.48 (0.04-2.96)	0% (0-0)
Zimbabwe	AM 25% Reduction	0.62 (0.42-0.85)	7.26% (6-8.31)	1.97 (0.06-3.97)	32.86% (31.29-34.18)
Zimbabwe	AM 50% Reduction	0.67 (0.44-0.92)	14.91% (12.19-17.21)	2.52 (0.07-5.13)	69.77% (65.73-73.18)
Zimbabwe	AM 75% Reduction	0.71 (0.47-1)	22.97% (18.58-26.71)	3.13 (0.09-6.44)	111.04% (103.46-117.48)
Zimbabwe	ITN 25% Reduction	0.58 (0.39-0.79)	0.07% (0.04-0.11)	1.49 (0.04-2.96)	0.07% (0.04-0.11)
Zimbabwe	ITN 50% Reduction	0.58 (0.39-0.79)	0.1% (0.06-0.14)	1.49 (0.04-2.96)	0.1% (0.06-0.14)
Zimbabwe	ITN 75% Reduction	0.58 (0.39-0.79)	0.2% (0.12-0.3)	1.49 (0.04-2.97)	0.2% (0.12-0.3)
Zimbabwe	Both 25% Reduction	0.62 (0.42-0.86)	7.33% (6.05-8.42)	1.97 (0.06-3.98)	32.95% (31.36-34.31)
Zimbabwe	Both 50% Reduction	0.67 (0.44-0.93)	15.02% (12.27-17.36)	2.52 (0.07-5.13)	69.93% (65.84-73.41)
Zimbabwe	Both 75% Reduction	0.71 (0.47-1)	23.2% (18.75-27.05)	3.14 (0.09-6.45)	111.43% (103.75-118.06)

Supplemental Methods

1.0 Expanded methodological description

This supplemental document describes our methods in greater detail than we provide in the main paper. To do so, we largely reproduce text from the supplemental information documents associated with our originally-published manuscripts, which are cited here as well as within the main text.

1.1 Plasmodium falciparum parasite rate (*Pf*PR)

The *Pf*PR results were generated using a geostatistical modelling approach, which produced pixel-level estimates for this metric, for each year, and for all endemic areas of sub-Saharan Africa. The response data for the model consisted of point-level measurements of parasite rate, typically gathered from children during household surveys, and then aggregated to the community level and affiliated with a cluster-level geographic location. In this analysis, we used 53,770 survey points that were collated within a database maintained by the Malaria Atlas Project[1]. These points were collected through surveys such as Malaria Indicator Surveys (MIS), which were conducted by groups such as the Demographic and Heath Surveys Program (DHS). All survey points were age-standardized to an age range of 2-10 years[2] prior to geostatistical modelling, and measurements collected via rapid diagnostics tests (RDT) were further calibrated to make them comparable with *Pf* test results derived using microscopy[3]. The predictor data consisted of environmental metrics and spatio-temporal estimates of malaria interventions. The environmental datasets were composite metrics that were correlated with malaria transmission, as identified using a thorough literature review, and later refined with an intensive variable selection process[4]. The geostatistical model was fit using integrated nested Laplace approximations[5]. This method allowed pixel values in areas with no point-level measurements to be estimated by leveraging both (1) the simple relationships between the response and predictor variables, and (2) spatial and temporal information present within the response data. In doing so, the geostatistical model utilized spatial and temporal autocorrelation to improve the resulting estimates of *Pf*PR. Note that the geostatistical model remained unchanged from its original formulation, as described in Bhatt et. Al (2015)[6], but the response and predictor datasets were updated considerably in the intervening years. The following text (sections 1.2-1.5) is taken directly from selected sections of the supplementary information for Bhatt et. al (2015)[6].

1.2 Latent Gaussian Process Model

The construction of malaria endemicity maps using model based geostatistics and/or latent Gaussian models[5,7,8] has been described in detail previously[9-11]. In brief, latent Gaussian modelling techniques utilise stochastic Gaussian processes to allow for extremely flexible extensions of generalised linear models[12] while still maintaining a rigorous probabilistic framework (i.e., Bayesian inference). Given temporally dynamic data characterizing environmental conditions (X), interventions ($\mathcal{I}$), baseline parasite rate ($\mathcal{B}$), and residual structural random effects (Z), the LGM model translates discrete survey *Pf*PR observations into a continuous model, $f(\cdot)$, of prevalence through space and time. The model is fully Bayesian and therefore combines information contained in the data (likelihood), with uncertainty in the data generating process (prior), to yield a posterior probability distribution that weights all the available evidence. From this continuous model we can evaluate the posterior predictive distribution by integrating over the posterior distribution with the parameter uncertainty, resulting in a prediction that spans all of Africa at an approximate 5 km by 5 km spatial resolution.

1.3 Latent Gaussian model formulation

To simplify notation and aid readability in this section we redefine $PfPr_{s,t,2-10} = y_{s,t}$ Given the n transformed and adjusted $g(y_{s,t}) = PfPr_{s,t,2-10}$ response observations we use a hierarchical LGM with covariates as environmental conditions ($X_{s,t}$), interventions ($\mathcal{I}_{s,t}$), baseline parasite rate ($\mathcal{B}_s$) and random components as *iid* country specific random effects (Y_c) and unobserved structural random effects ($Z_{s,t}$). At a set of prediction locations ($x_{s,t}$), the model can be expressed through the measurement equation:

$$g^{-1}(x_{s,t}) \sim \beta X_{s,t} + \alpha \mathcal{I}_{s,t} + \gamma \mathcal{B}_s + Z_{s,t} + Y_c + \epsilon \quad (1)$$

Where $g(\cdot)$ is the empirical logit[12] and $\epsilon \sim N(0, I\sigma_e^2)$ is the measurement error which is both spatially and temporally uncorrelated. Our response variables are dichotomous random variables, for which the canonical likelihood function is the binomial. Unfortunately models fitted here using a binomial likelihood function grossly misspecified the posterior variance, and the posterior credible intervals did not faithfully reflect the underlying uncertainty in the data (determined from plots of probability integral transforms and Bayesian coverage). We tried

overdispersed models (beta- binomial) and zero inflated models to correct the posterior variance, but these also failed to produce a well-specified model. We therefore opted to use a Gaussian likelihood, where the data was transformed through an empirical logit[7,13] ($g(\cdot)$), which produced well specified posteriors both in terms of calibration of the variance and out of sample prediction of the mean.

We use a Gaussian process prior on the mean of our Gaussian likelihood, parameterised as a Gaussian Markov random field[14] (GMRF). This Gaussian process allowed for highly flexible stochastic realisations, incorporation of spatio-temporal correlation, and extremely favourable computational properties.

The hyper parameters (θ) that parameterise the likelihood and GMRF prior were defined using probability distributions thus completing the Bayesian hierarchical model formulation.

By combining the Gaussian likelihood, the GMRF prior and the parameter hyperpriors, the hierarchical model was defined as

$$\theta \sim \pi(\theta) \quad (2)$$

$$(x_{s,t} \mid \theta) \sim Gaussian\ (\mu_{s,t|\theta}, Q^{-1}_{x_{s,t}\ |\theta}) \quad (3)$$

$$(y_{s,t} \mid x_{s,t}, \theta) \sim Gaussian\ (Ax_{s,t} + Y_c, Q^{-1}_{y_{s,t}|x_{s,t},\theta}) \quad (4)$$

where $\mu_{s,t|\theta} = \beta X_{s,t} + \alpha \mathcal{I}_{s,t} + \gamma \mathcal{B}_s + Y_c$, $\theta \in [\sigma_e, \sigma_{iid}, \beta, \alpha, \gamma, \tau, \kappa]$ is a vector of prior probability distributions on the hyper parameters, A is a sparse observation matrix that maps the GMRF to function evaluations at local observations and $Q^{-1}_{y_{s,t}|x_{s,t},\theta} = I\ {}^{1}\!/_{\sigma_e^2}$ where I is the identity matrix.

Given the properties of Gaussian distributions[8] we were able to define in closed form the conditional distribution of predictions given the data and hyper parameters:

$$g^{-1}(x_{s,t} \mid y_{s,t}, \theta) \sim Gaussian(\mu_{s,t|\theta} + Q^{-1}_{x_{s,t}|y_{s,t},\theta} A^T Q_{y_{s,t}|x_{s,t},\theta}(y_{s,t} - A\mu_{s,t|\theta}), Q^{-1}_{x_{s,t}|y_{s,t},\theta}) \quad (4)$$

where $Q^{-1}_{x_{s,t}|y_{s,t},\theta} = Q_{x_{s,t|\theta}} + A^T Q_{y_{s,t}|x_{s,t},\theta} A$. Equation 4 provides the conditional expectation or mean for a prediction location given the data and hyper parameters. Given the GMRF prior the conditional in equation 4 is also sparse[15] which allows us to sample individual realisations in a computationally efficient manner thereby allowing for aggregations to different scales, while keeping the correct marginal variances[16].

1.3.1 $\boldsymbol{\beta X_{s,t}}$ - The covariate component

Here $X_{s,t}$ is a matrix of 20 by n covariates intersected for each $y_{s,t}$ observation, and β is a coefficient vector. The selection of these covariates is described briefly in section 3 and readers are directed to Weiss et al 2015[4] for a complete description.

1.3.2 $\boldsymbol{Z_{s,t}}$ - The spatio-temporal covariance function for structured random effects

$Z_{s,t}$ can be thought of as a state process, and is assumed to be a spatio-temporal Gaussian process that is correlated in space by a Matérn covariance function, and in time by first order autoregressive dynamics. The Matérn spatial component is represented through a GMRF as

$$w_s \sim N(0, Q_s) \quad (5)$$

where the sparse precision matrix Q_s is the sparse finite element solution to the stochastic partial differential equation[15]

$$(k^2 - \Delta)^{\frac{\alpha}{2}}(\tau x(s)) = \varepsilon(s) \quad (6)$$

where Δ is the Laplacian, k is the spatial scale/range parameter, τ controls the variance, α is the spatial smoothness parameter (fixed at $\alpha = 2$), and $\varepsilon(s)$ is the spatial white noise process. s is defined on a spherical manifold in Cartesian $\mathbb{R}^3$. For more details see Lindgren et al 2011[15].

Temporal correlation is modelled by first order auto regressive dynamics as $w_t = \phi w_{t-1}$, which yields the GMRF $w_t \sim N(0, Q_t)$.

Combining these two GMRFs into a joint spatio-temporal field is achieved through a Kronecker product of the spatial and temporal precision matrices: $Q_{s,t} = Q_t \otimes Q_s$ where $Q_{s,t}$ is a spatio-temporal precision matrix[17]. The spatio-temporal GMRF prior in the measurement equation is therefore defined as $Z_{s,t} \sim Gaussian(0, Q_{s,t})$.

1.3.3 $\gamma\mathcal{B}_s$ - the baseline parasite rate component

The idea of evaluating the baseline parasite rate was to establish a "fundamental niche" of malaria prevalence that characterizes the stable parasite rate across all of Africa given no interventions or changes in environmental and socioeconomic conditions. This approach has been well established in disease ecology literature[18-20]. The baseline parasite rate is crucial for estimating the change in parasite rate through time, as the baseline parasite rate should equal the current parasite rate in the absence of other changes (e.g., changing climatic patterns, anti-malarial interventions, or unobserved but correlated changes). This is reflected in the measurement equation $\Phi^{-1}(p_i^{MIC}) = -0.24 + 0.95 * \Phi^{-1}(p_i^{RDT})$ where the current parasite rate at any time in space is the same as the baseline parasite rate for a given geographic location given no change in the terms associated with temporally varying covariates (shown in red).

$$g^{-1}(y_{s,t}) \sim \beta X_{s,t} + \alpha \mathcal{I}_{s,t} + \gamma \mathcal{B}_s + Z_{s,t} + Y_c + \epsilon \quad (7)$$

Given this conceptual dependency in the measurement equation, deviations of the parasite rate are then described through the (1) environmental and socio-economic covariates, which determine fluctuations (both seasonally and long term) through time, (2) interventions (ITN, IRS, and ACT), and (3) unobserved correlated temporal and spatial patterns (determined from the spatio-temporal covariance).

To model the baseline parasite rate $\mathcal{B}_s$ we split the entire $y_{s,t}$ dataset, and created a subset of k observations that occurred at spatio-temporal locations with minimal intervention coverage yet high *Pf* (i.e., ITN use less than 7% and an IRS population at risk coverage less than 30%). The k subset was then used to define a purely spatial model of *Pf*PR that encapsulated inter- and intra-annual environmental conditions overly many years, thus producing a model for estimating *Pf*PR that respects natural influences on *Pf*PR while masking the influence of interventions. The application of this model produced a synoptic *Pf*PR surface that effectively serves as a counterfactual dataset for the state of *Pf*PR in the absence of ITN, IRS, and ACT use. The measurement equation used was:

$$\mathcal{B}_s \sim \beta X_s + Z_s + \epsilon \quad (8)$$

where X_s are the environmental covariates for locations s in the year 2000, and Z_s is a Matérn GMRF. Out of sample validation and visual inspection showed a large degree of robustness to these thresholds (within sensible variations). It should be noted that the fitting of the baseline model was done separately from the main parasite rate model. This was done primarily for computational tractability and ease of implementation. We did compare the jointly fitted baseline and main parasite rate model against the separate model and found the results to be nearly identical.

1.3.4 Prior probability specifications

Prior probabilities on the fixed effect coefficients were specified as Normally distributed

$$\beta, \alpha, \gamma \sim \text{Gaussian } (0,1000) \quad (8)$$

Prior probabilities on the Matérn range and variance parameters were distributed on a log scale using normal distribution as:

$$\log(\tau) \sim \text{Gaussian } (0,10) \quad (9)$$

$$\log(\kappa) \sim \text{Gaussian } (0,1) \quad (10)$$

The autoregressive parameter for the temporal covariance was distributed on a log transformed proportional scale as:

$$\log((1+\phi)/(1-\phi)) \sim \text{Gaussian } (0,0.15) \quad (11)$$

The country specific random effects variance and the Gaussian likelihood variance were both independently distributed log scale by a log gamma distribution:

$$\log\left(\frac{1}{\sigma_{iid}^2}\right), \log\left(\frac{1}{\sigma_e^2}\right) \sim Loggamma(1,5^{-5}) \quad (12)$$

Changes to the prior distributions did not significantly change posterior fits and specifications using different prior distributions produced nearly the same posterior parameter fits, showing a high degree of robustness to prior specifications.

1.4 Fitting and validation of the LGM

Fitting the LGM was done using an integrated Laplace approximation (INLA) of the posterior conducted using the R package INLA[5]. Testing of the INLA algorithm was performed by comparison on smaller subsets fitted using Hamiltonian Markov Chain Monte Carlo (HMCMC[21]), which showed almost exact correspondence.

Posterior validation was performed by calculating cross validation probability integral transforms (PIT) and five-fold out of sample cross validation. PIT was defined as:

$$PIT_i = \int \mathbb{P}(Y_i \leq PfPr_i | PfPr_{-i}, \theta)\mathbb{P}(\theta | PfPr_{-i})\mathrm{d}\theta \tag{13}$$

The PIT in Equation 13 links actual PfPr observations relative to the percentiles of their predicted distributions. Given a set of observations $x_1, .., x_n$ with corresponding cumulative predictive distributions $F_1, .., F_n$, the empirical distribution of $F_1(x_1), .., F_n(x_n)$ can be compared against a unit uniform distribution. Therefore, if a sequence of predictive densities coincides with the true data generating process the PITs will be distributed as a unit uniform distribution[22]. Essentially the PIT asks the question "could the fitted model reproduce the data?" Further information on the calculation of the PIT in the INLA framework are describe elsewhere[23]. It should be noted that in practice the PIT values are not always exactly uniform, although convergence is nearly assured as the sample size tends to infinity. Therefore, odd PIT values resulting in u-shaped, nshaped, or skewed distributions point to a miscalibrated posterior where as those which are approximately uniform point to a well-calibrated posterior with good Bayesian coverage.

In addition to calculating PIT values (figure 4) which provide a rigorous evaluation of the posterior predictive distribution we also performed conventional five-fold out of sample validation[24]. In accordance with current best practices[25] we randomly partitioned our data into five non overlapping subsets (testing sets) and ran five models on the remaining data (training sets). We then evaluated the correlation and coefficient of determination (R-squared), the mean absolute error, and the mean squared error. Where we had cross sectional national surveys we also compared the average predicted parasite rate to the actual survey rate. The correlation and coefficient of determination were 0.87 and 0.75 respectively and the mean absolute error and mean squared error were 9% and 2% respectively. These values indicated a high degree of out of sample predictive accuracy.

1.4 Conditional simulations

Realisations from the fitted LGM were created using the conditional distribution in Equation 12. For a given set of joint parameters $\theta_k \in [\beta, \alpha, \gamma, \tau, \kappa, \phi, \sigma^2_{iid}, ' sigma^2_e]$ a realization from the LGM is evaluated from the conditional distribution as

$$g^{-1}(x_{s,t} | y_{s,t}, \theta)_k = \frac{\sum_{l \in [1,..,12]} Gaussian(\mu_{s,t,l|\theta_k} + Q^{-1}_{x_{s,t}|y_{s,t},\theta_k} A^T Q_{y_{s,t}|x_{s,t},\theta_k}(y_{s,t} - A\mu_{s,t|\theta_k}), Q^{-1}_{x_{s,t}|y_{s,t},\theta_k})}{\sum_{l \in [1,..,12]} l} \tag{14}$$

Where *l* is the month, which is used to incorporate the monthly variation in the dynamic covariates when predicting at a yearly interval *t*. To incorporate the temporal uncertainty, all years were predicted simultaneously providing a full conditional realization across the entire spatio-temporal range. In total we generated $k = [1, ..100]$ samples across the whole spatio-temporal range.

2.0 Prevalence to incidence conversion

We model incidence from *Pf*PR and, as with the *Pf*PR, a description of this model was included in the supplemental information for Bhatt et. al (2015)[6], which we reproduce below. For more details, please see the original publication by Cameron et. al (2015)[26].

2.1 Ensemble Model for Clinical Incidence

Given the prohibitive cost of active case detection (ACD) as a means to directly monitor the clinical incidence rate of Plasmodium falciparum malaria on large scales, recent efforts towards burden enumeration have focussed on identifying a functional relationship between 2-10 y/o prevalence and population-wide incidence as a means to transform cartographic prevalence surfaces to approximate incidence maps[9,27,28]. The latest generation of micro-simulation models, in which entomological inoculation and the course of infection are followed stochastically at the level of individual hosts, offer a sophisticated means to infer this relationship subject to our current epidemiological understanding of the malaria parasite. As no consensus yet exists as to the exact model structure required to accurately represent the observed transmission dynamics, we developed an ensemble modelling approach to constrain the prevalence-incidence relationship with a combination of three contemporary micro-simulation codes (OpenMalaria[29,30], the EMOD DTK[31,32], and an in-house version of the Griffin et al. model[33,34]) fit against a purpose-built empirical dataset of 26 historic ACD surveys covering a total of 30 unique sites across sub-Saharan Africa[35].

To render the posterior calibration procedure computationally tractable, a functional regression-based model emulator for each micro-simulation code was constructed from a library of 100,000 "noisy" (small population) simulation outputs. That is, we use the Nadaraya-Watson form[36] to predict the 2-10 y/o prevalence,

PfPR2-10 , and age-incidence curve, $I\ a$, belonging to a given input pairing of the Entomological inoculation rate (EIR) seasonality profile, $E(t)$, and model parameter list, θ:

$$\hat{R}[\theta, E(t)](a) = \frac{\sum_{i=1}^{n}[Pf\text{PR}_{2\text{-}10}, I(a)]K(d([\theta, E(t)]_i, [\theta, E(t)])/h)}{\sum_{i=1}^{n}K(d([\theta, E(t)]_i, [\theta, E(t)])/h)} \quad (15)$$

where $d(\cdot,\cdot)$ represents a suitable metric distance between points in the functional input space, $K(\cdot)$ denotes a positive definite weighting kernel, and h the associated bandwidth. Simulation from the emulated posterior given our observational dataset was achieved via a pseudo-marginal MCMC sampling scheme[37] with a negative binomial likelihood function adopted to allow for over-dispersion in the observed incidence counts in addition to a survey-specific random effects term.

Age-, seasonality-, and treatment-structured prevalence-incidence curves were drawn from the posterior predictive distributions of each calibrated emulator and combined into a single ensemble model using a bespoke weighting scheme based on the M-posteriors algorithm[38]. The latter, in which two- and three-way agreements between calibrated models are automatically favoured, being preferred over either Bayesian model averaging (overly prior-sensitive) or a raw uniform combination (overly conservative) for our purposes. With the six way structuring of prevalence-incidence curves so produced we seek to improve on the precision of our burden estimates through a stratified sampling approach taking into account the available information concerning variations in the local demography, transmission conditions, and standards of medical service provision across the African continent.

3.0 Mortality in Africa

Mortality estimates were created by first cleaving, at the pixel-level, incidence counts into treated and untreated cases. Cases were categorized as treated or untreated using the effective treatment with an antimalarial estimates, with untreated cases susceptible to death using a pixel-level case fatality rate (CFR). CFR was modeled using cause of death (CoD) data extracted from the Global Burden of Disease (GBD) project and consists of a mixture of verbal and formal autopsy data, both of this are typically acquired at clinics and may be aggregated by administrative units. A full description of this method is provided in Gething et al. (2016)[39], and the following text is reproduced from a follow-on paper that also utilized this methodology[40].

3.1 Effective treatment

Effective treatment was estimated by estimating the usage of three antimalarial drugs: chloroquine (CQ), sulfadoxine-pyrimethamine (SP) and artemisinin combination therapy (ACT). This was combined with estimates of drug resistance to give a spatiotemporal cube of effective treatment rate. To disaggregate into GBD age-bins, we separately ran a traditional national-level CODEm model with covariates: prevalence of P. falciparum in 2–10 age group, P. falciparum incidence rate, years of education, access to effective antimalarial drugs, and health system access. The effective treatment rate was combined with the incidence rate cube to derive a third cube estimating the incidence of untreated cases. Details of the models can be found in the appendix to Gething et al.[39] .

3.2 Case fatality rate

For each site-year for which CoD malaria cause fraction data were available we (i) estimated a site-year specific malaria mortality rate, as the product of malaria cause fraction and all-cause mortality rate (with the latter drawn from national-level values); (ii) divided the malaria mortality rate by the site-year specific estimate of untreated malaria incidence rate to estimate a site-year specific case fatality rate (CFR) amongst untreated malaria cases. These derived site-year specific CFR values were then used in a mixed- effects regression model to estimate pixel-year CFR for each 5km × 5km grid cell. The covariates used in the model were the log of country-year all-cause mortality, night-time lights, accessibility and fractional land- cover classes, and study-specific age and sex, with the location of each study site included as a national- level random effect. Data were weighted by sample size (i.e. the number of all-cause deaths observed in each study site-year). Further details can be found in previous publications.[39]

3.3 Cartographic mortality cube

To estimate the fatal burden of P. falciparum malaria in Africa, we used epidemiologic measures of non-fatal malaria burden as described in section "Africa prevalence model". Pixel-year predictions of CFR were then multiplied by the corresponding untreated incidence rate cube to yield a pixel-year mortality rate estimate, which was then multiplied by pixel-year population to compute pixel-year malaria death estimates. These were then aggregated to yield the required GBD national or subnational death estimates.

4.0 References

1 Guerra, C. A. *et al.* Assembling a global database of malaria parasite prevalence for the Malaria Atlas Project. *Malaria Journal* **6**, 17-17, doi:10.1186/1475-2875-6-17 (2007).

2 Smith, D. L., Guerra, C. A., Snow, R. W. & Hay, S. I. Standardizing estimates of the Plasmodium falciparum parasite rate. *Malaria Journal* **6**, 131-131, doi:10.1186/1475-2875-6-131 (2007).

3 Mappin, B. *et al.* Standardizing Plasmodium falciparum infection prevalence measured via microscopy versus rapid diagnostic test. *Malaria Journal* **14**, 460, doi:10.1186/s12936-015-0984-9 (2015).

4 Weiss, D. J. *et al.* Re-examining environmental correlates of Plasmodium falciparum malaria endemicity: a data-intensive variable selection approach. *Malaria journal* **14**, 68 (2015).

5 Rue, H., Martino, S. & Chopin, N. Approximate Bayesian inference for latent Gaussian models by using integrated nested Laplace approximations. *Journal of the royal statistical society: Series b (statistical methodology)* **71**, 319-392 (2009).

6 Bhatt, S. *et al.* The effect of malaria control on Plasmodium falciparum in Africa between 2000 and 2015. *Nature* (2015).

7 Diggle, P. J., Tawn, J. A. & Moyeed, R. A. Model-based geostatistics. *Journal of the Royal Statistical Society Series C-Applied Statistics* **47**, 299-326 (1998).

8 Rasmussen, C. E. in *Advanced lectures on machine learning* 63-71 (Springer, 2004).

9 Hay, S. I. *et al.* A world malaria map: Plasmodium falciparum endemicity in 2007. *PLoS Med* **6**, e1000048 (2009).

10 Gething, P. W. *et al.* A new world malaria map: Plasmodium falciparum endemicity in 2010. *Malaria Journal* **10**, 378 (2011).

11 Gething, P. W. *et al.* Climate change and the global malaria recession. *Nature* **465**, 342-345 (2010).

12 McCullagh, P. *Generalized linear models*. (Routledge, 2018).

13 Stanton, M. C. & Diggle, P. J. Geostatistical analysis of binomial data: generalised linear or transformed Gaussian modelling? *Environmetrics* **24**, 158-171 (2013).

14 Rue, H. & Held, L. *Gaussian Markov random fields: theory and applications*. (CRC press, 2005).

15 Lindgren, F., Rue, H. & Lindström, J. An explicit link between Gaussian fields and Gaussian Markov random fields: the stochastic partial differential equation approach. *Journal of the Royal Statistical Society: Series B (Statistical Methodology)* **73**, 423-498 (2011).

16 Gething, P. W., Patil, A. P. & Hay, S. I. Quantifying aggregated uncertainty in Plasmodium falciparum malaria prevalence and populations at risk via efficient space-time geostatistical joint simulation. *PLoS Comput. Biol.* **6** (2010).

17 Cameletti, M., Ignaccolo, R. & Bande, S. Comparing spatio-temporal models for particulate matter in Piemonte. *Environmetrics* **22**, 985-996 (2011).

18 Bhatt, S. *et al.* The global distribution and burden of dengue. *Nature* **496**, 504-507 (2013).

19 Stevens, K. B. & Pfeiffer, D. U. Spatial modelling of disease using data-and knowledge-driven approaches. *Spatial and spatio-temporal epidemiology* **2**, 125-133 (2011).

20 Elith*, J. *et al.* Novel methods improve prediction of species' distributions from occurrence data. *Ecography* **29**, 129-151 (2006).

21 Carpenter, B. *et al.* Stan: A probabilistic programming language. *Journal of statistical software* **76** (2017).

22 Gneiting, T., Balabdaoui, F. & Raftery, A. E. Probabilistic forecasts, calibration and sharpness. *Journal of the Royal Statistical Society: Series B (Statistical Methodology)* **69**, 243-268 (2007).

23 Held, L., Schrödle, B. & Rue, H. in *Statistical modelling and regression structures* 91-110 (Springer, 2010).

24 Bishop, C. M. *Pattern recognition and machine learning*. (springer, 2006).

25 Abu-Mostafa, Y. S., Magdon-Ismail, M. & Lin, H.-T. *Learning from data*. Vol. 4 (AMLBook New York, NY, USA:, 2012).

26 Cameron, E. *et al.* Defining the relationship between infection prevalence and clinical incidence of Plasmodium falciparum malaria. *Nature Communications* **6** (2015).

27 Snow, R. W., Guerra, C. A., Noor, A. M., Myint, H. Y. & Hay, S. I. The global distribution of clinical episodes of Plasmodium falciparum malaria. *Nature* **434**, 214-217 (2005).

28 Patil, A. P. *et al.* Defining the relationship between Plasmodium falciparum parasite rate and clinical disease: statistical models for disease burden estimation. *Malaria journal* **8**, 186 (2009).

29 Smith, T. *et al.* Mathematical modeling of the impact of malaria vaccines on the clinical epidemiology and natural history of Plasmodium falciparum malaria: Overview. *The American journal of tropical medicine and hygiene* **75**, 1-10 (2006).
30 Smith, T. *et al.* Ensemble modeling of the likely public health impact of a pre-erythrocytic malaria vaccine. *PLoS Med* **9**, e1001157 (2012).
31 Eckhoff, P. A. A malaria transmission-directed model of mosquito life cycle and ecology. *Malaria journal* **10**, 303 (2011).
32 Wenger, E. A. & Eckhoff, P. A. A mathematical model of the impact of present and future malaria vaccines. *Malaria Journal* **12**, 1-13 (2013).
33 Griffin, J. T., Ferguson, N. M. & Ghani, A. C. Estimates of the changing age-burden of Plasmodium falciparum malaria disease in sub-Saharan Africa. *Nature communications* **5**, 1-10 (2014).
34 Griffin, J. T. *et al.* Reducing Plasmodium falciparum malaria transmission in Africa: a model-based evaluation of intervention strategies. *PLoS Med* **7**, e1000324 (2010).
35 Battle, K. E. *et al.* Global database of matched Plasmodium falciparum and P. vivax incidence and prevalence records from 1985–2013. *Scientific Data* **2**, 1-12 (2015).
36 Ferraty, F., Van Keilegom, I. & Vieu, P. Regression when both response and predictor are functions. *Journal of Multivariate Analysis* **109**, 10-28 (2012).
37 Andrieu, C. & Roberts, G. O. The pseudo-marginal approach for efficient Monte Carlo computations. *The Annals of Statistics* **37**, 697-725 (2009).
38 Minsker, S., Srivastava, S., Lin, L. & Dunson, D. in *International conference on machine learning.* 1656-1664.
39 Gething, P. W. *et al.* Mapping Plasmodium falciparum Mortality in Africa between 1990 and 2015. *New England Journal of Medicine* **375**, 2435-2445 (2016).
40 Weiss, D. J. *et al.* Mapping the global prevalence, incidence, and mortality of *Plasmodium falciparum*, 2000-17: a spatial and temporal modelling study. *The Lancet*, doi:10.1016/S0140-6736(19)31097-9 (2019).
